# Absence of the ER Cation Channel *TMEM38B*/TRIC-B Disrupts Intracellular Calcium Homeostasis and Dysregulates Collagen Synthesis in Recessive Osteogenesis Imperfecta

**DOI:** 10.1371/journal.pgen.1006156

**Published:** 2016-07-21

**Authors:** Wayne A. Cabral, Masaki Ishikawa, Matthias Garten, Elena N. Makareeva, Brandi M. Sargent, MaryAnn Weis, Aileen M. Barnes, Emma A. Webb, Nicholas J. Shaw, Leena Ala-Kokko, Felicitas L. Lacbawan, Wolfgang Högler, Sergey Leikin, Paul S. Blank, Joshua Zimmerberg, David R. Eyre, Yoshihiko Yamada, Joan C. Marini

**Affiliations:** 1 Section on Heritable Disorders of Bone and Extracellular Matrix, NICHD, NIH, Bethesda, Maryland, United States of America; 2 Molecular Biology Section, NIDCR, NIH, Bethesda, Maryland, United States of America; 3 Section on Integrative Biophysics, NICHD, NIH, Bethesda, Maryland, United States of America; 4 Section on Physical Biochemistry, NICHD, NIH, Bethesda, Maryland, United States of America; 5 Department of Orthopaedics and Sports Medicine, University of Washington, Seattle, Washington, United States of America; 6 School of Clinical and Experimental Medicine, Institute of Biomedical Research, University of Birmingham, Birmingham, United Kingdom; 7 Department of Endocrinology and Diabetes, Birmingham Children’s Hospital, Birmingham, United Kingdom; 8 Connective Tissue Gene Tests, Allentown, Pennsylvania, United States of America; 9 Department of Medical Genetics, Children’s National Medical Center, Washington D.C., United States of America; Murdoch Childrens Research Institute, AUSTRALIA

## Abstract

Recessive osteogenesis imperfecta (OI) is caused by defects in proteins involved in post-translational interactions with type I collagen. Recently, a novel form of moderately severe OI caused by null mutations in *TMEM38B* was identified. *TMEM38B* encodes the ER membrane monovalent cation channel, TRIC-B, proposed to counterbalance IP_3_R-mediated Ca^2+^ release from intracellular stores. The molecular mechanisms by which *TMEM38B* mutations cause OI are unknown. We identified 3 probands with recessive defects in *TMEM38B*. TRIC-B protein is undetectable in proband fibroblasts and osteoblasts, although reduced *TMEM38B* transcripts are present. TRIC-B deficiency causes impaired release of ER luminal Ca^2+^, associated with deficient store-operated calcium entry, although SERCA and IP_3_R have normal stability. Notably, steady state ER Ca^2+^ is unchanged in TRIC-B deficiency, supporting a role for TRIC-B in the kinetics of ER calcium depletion and recovery. The disturbed Ca^2+^ flux causes ER stress and increased BiP, and dysregulates synthesis of proband type I collagen at multiple steps. Collagen helical lysine hydroxylation is reduced, while telopeptide hydroxylation is increased, despite increased LH1 and decreased Ca^2+^-dependent FKBP65, respectively. Although PDI levels are maintained, procollagen chain assembly is delayed in proband cells. The resulting misfolded collagen is substantially retained in TRIC-B null cells, consistent with a 50–70% reduction in secreted collagen. Lower-stability forms of collagen that elude proteasomal degradation are not incorporated into extracellular matrix, which contains only normal stability collagen, resulting in matrix insufficiency. These data support a role for TRIC-B in intracellular Ca^2+^ homeostasis, and demonstrate that absence of *TMEM38B* causes OI by dysregulation of calcium flux kinetics in the ER, impacting multiple collagen-specific chaperones and modifying enzymes.

## Author Summary

Osteogenesis imperfecta (OI) is a heritable disorder of connective tissues characterized by fracture susceptibility and growth deficiency. Most OI cases are caused by autosomal dominant mutations in the genes encoding type I collagen, *COL1A1* and *COL1A2*. Delineation of novel gene defects causing dominant and recessive forms of OI has led to the understanding that the bone pathology results not only from abnormalities in type I collagen quantity and primary structure, but also from defects in post-translational modification, folding, intracellular transport and extracellular matrix incorporation. Recently, mutations in *TMEM38B*, which encodes the integral ER membrane K^+^ channel TRIC-B, have been identified as causative for the OI phenotype. However, the mechanism by which absence of TRIC-B causes OI has not been reported. Using cell lines established from three independent probands, we have demonstrated that absence of TRIC-B leads to abnormal ER Ca^2+^ flux and store-operated calcium entry (SOCE), although ER steady state Ca^2+^ is normal. Disruption of intracellular calcium dynamics alters the expression and activity of multiple collagen interacting chaperones and modifying enzymes within the ER. Thus TRIC-B deficiency causes OI by dysregulation of collagen synthesis, through the impairment of calcium-dependent gene expression and protein-protein interactions within the ER.

## Introduction

Osteogenesis imperfecta (OI), also known as “brittle bone disease”, is a heritable disorder of connective tissue characterized by short stature, bone deformity, and susceptibility to fracture from minimal trauma due to reduced strength and increased brittleness of bone [1]. OI occurs in approximately 1 in 15,000–20,000 births and ranges in severity from a sub-clinical, osteopenic phenotype to perinatal lethality, as described by the Sillence Classification [2]. The majority of cases (85%) result from dominant mutations in the genes that encode type I collagen, *COL1A1* and *COL1A2*. The less prevalent forms of OI, which mostly have recessive inheritance, are caused by mutations in genes whose products are involved in co- and post-translational interactions with type I collagen [3]. Although defects in more than 17 genes have now been implicated as causative for the OI phenotype, the underlying bone pathology results from abnormalities in type I collagen quantity, primary structure, post-translational modification, folding, intracellular transport and extracellular matrix incorporation, thereby delineating OI as a collagen-related bone disorder [4].

Type I collagen is the major component of the scaffolding of the extracellular matrix of skin, tendon and bone [5]. It is synthesized by fibroblasts and osteoblasts as a heterotrimer containing a central helical region consisting of 338 uninterrupted repeats of the Gly-Xaa-Yaa triplet, flanked by globular domains at each terminus. Biosynthesis of procollagen is a complex process that requires several co- and post-translational modifications within the endoplasmic reticulum (ER), including formation of inter- and intra-chain disulfide bonds within the C-terminal globular region, isomerization of peptidyl-prolyl bonds, hydroxylation of multiple helical lysyl and prolyl residues by lysyl hydroxylase (LH1) and prolyl 4-hydroxylase (P4H), respectively, and glycosylation of some hydroxylysines [6]. In addition, two specific proline residues are modified by the prolyl 3-hydroxylase (P3H) complex [7,8]. The post-translational modifications occur before, and to a major extent enable, proper collagen helical folding. Upon secretion from the cell, the globular domains are removed by specific pericellular propeptidases, followed by incorporation of the mature collagen molecule into heterotypic fibrils that are stabilized by lysine-derived intermolecular crosslinks [9–11].

Synthesis of type I collagen is a calcium-regulated process. Ca^2+^ binding to the C-terminal globular domain stabilizes interchain hydrogen and disulfide bonds required for procollagen trimerization and folding [12]. Furthermore, the activities of several Ca^2+^-binding chaperones, foldases and modifying enzymes in the ER may be regulated by fluctuations in lumenal [Ca^2+^], which are mediated through calcium release by inositol triphosphate receptors (IP_3_R) or ryanodine receptors (RyR), and calcium uptake by the sarcoplasmic-ER Ca^2+^-ATPase type 2b (SERCA2b) [13–15]. IP_3_R activity may also be linked to the regulation of collagen expression via [Ca^2+^]_i_ signaling pathways [16–18], while the ER membrane translocon complex component, TRAM2, is suggested to couple SERCA2b activity with translation of type I procollagen chains [19]. Thus, disruption of ER Ca^2+^ homeostasis has the potential to alter the expression and activities of multiple collagen interacting proteins and dysregulate the process of collagen biosynthesis.

Recently a novel form of recessive OI, caused by a founder mutation, was reported in Bedouin families from Israel and Saudi Arabia [20,21]. The probands from these families presented with a moderately severe bone phenotype, characterized as type XIV OI (OMIM #615066), with bowing and fragility of long bones leading to multiple fractures in infancy. Two groups independently identified the same mutation in *TMEM38B*, a deletion of exon 4 and surrounding intronic sequence [20,21]. A second deletion mutation was identified in an Albanian child, born to consanguineous parents, who harboured a genomic deletion including exons 1 and 2 of *TMEM38B* [22]. Homozygosity for two point mutations in *TMEM38B* was recently reported in three probands from non-consanguineous Han Chinese families, including a splice acceptor site variant in intron 3 and a nonsense mutation in exon 4 [23].

*TMEM38B* encodes the ER membrane TRimeric Intracellular Cation channel subtype B (TRIC-B), a ubiquitous protein that functions as a monovalent cation channel. The TRIC-B channel has been proposed to affect Ca^2+^ homeostasis in the ER, the major site of intracellular Ca^2+^ storage [24]. Although *TMEM38B* null mutations were demonstrated to be the genetic cause of moderately severe bone dysplasia, the molecular mechanisms through which absence of TRIC-B causes an OI phenotype are unknown. We identified three additional probands with *TMEM38B* mutations and investigated the effects of absence of TRIC-B on intracellular [Ca^2+^] flux and collagen biosynthesis. Our findings demonstrate that TRIC-B deficiency dysregulates multiple steps in collagen biosynthesis, placing the mechanism of TRIC-B absence within the collagen-related paradigm of OI.

## Results

### Mutations in *TMEM38B* cause recessively inherited Osteogenesis Imperfecta

**Fig 1:**
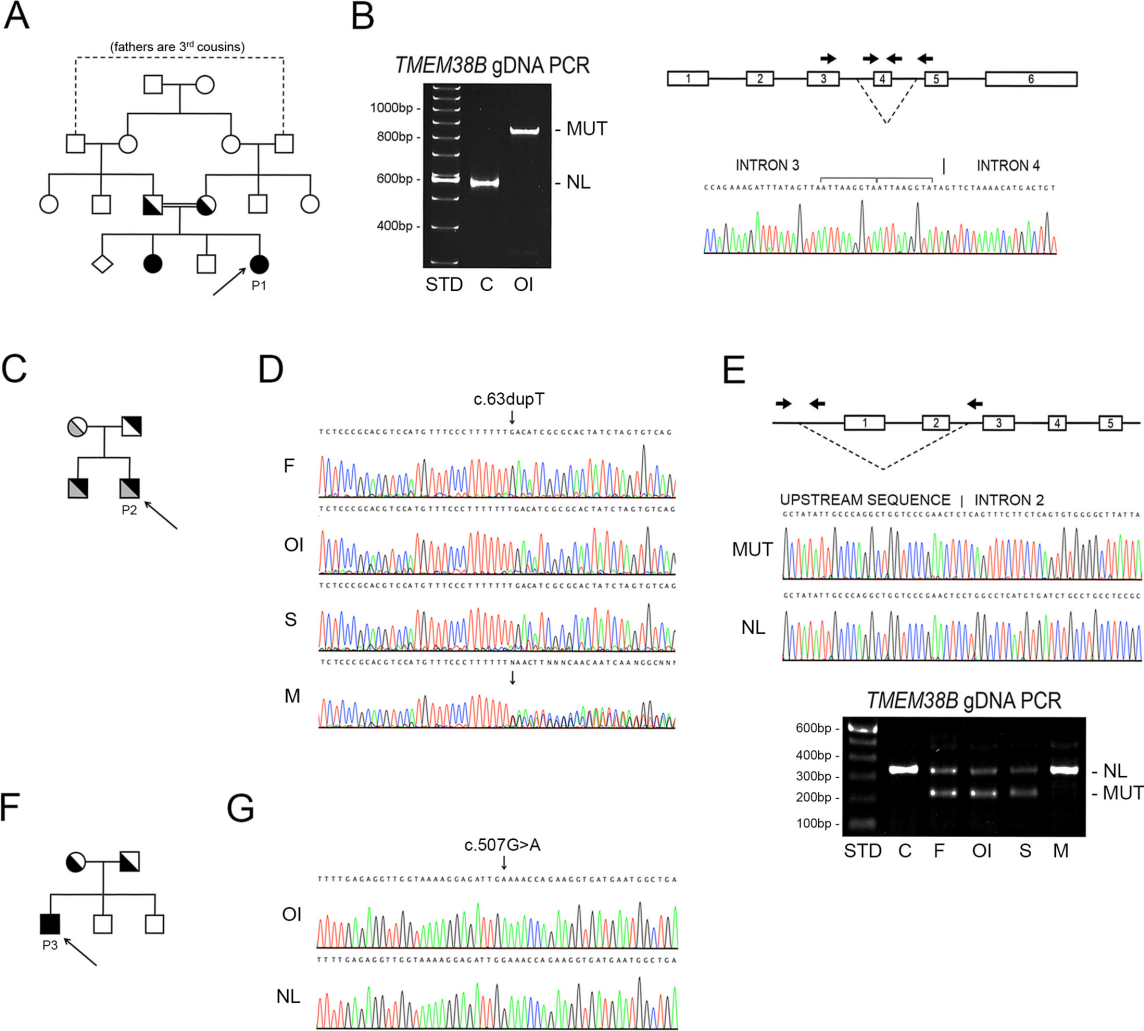
*TMEM38B* mutations cause recessive Osteogenesis imperfecta. **(A)** Pedigree of a four-generation consanguineous Saudi family, including Proband 1 (P1, arrow) and her affected sibling diagnosed with moderate OI (Type IV). **(B)** PCR amplification of genomic DNA using allele-specific primers (arrows) located adjacent to and within the previously identified *TMEM38B* deletion (dashed lines) in Bedouin pedigrees. Control genomic DNA (C) generated the expected 499 bp fragment (NL). In contrast, amplification of proband genomic DNA (OI) produced only a single 821 bp product (MUT), consistent with homozygosity for the Bedouin founder mutation, and confirmed by sequencing. **(C)** Family pedigree of Proband 2 (P2, arrow) and his affected sibling. **(D)** Sequence of *TMEM38B* exon 1 demonstrates apparent homozygosity in P2 (OI) and his affected sibling (S) for the maternal allele (c.63dupT mutation). While the mother (M) was heterozygous for the single nucleotide duplication, only the normal sequence was identified in the father (F). **(E)** The paternal allele in P2 is identical to a previously identified 35 Kb deletion encompassing the first two exons of *TMEM38B*. Heminested PCR of genomic DNA using allele-specific primers (arrows) confirmed the father (F), P2 (OI) and his sibling (S) as heterozygous for the paternal allele (MUT). **(F)** Pedigree of consanguineous Pakistani family including Proband 3 (P3) **(G)** Sequences of *TMEM38B* exon 4 using genomic DNA of P3 (OI) and normal control DNA (NL) demonstrates a homozygous nonsense mutation (c.507G>A).

Proband 1 (P1), a 20-month old female, was the second child with moderately severe OI born to apparently healthy consanguineous parents from Saudi Arabia (Fig 1A). Since a recurring deletion mutation of exon 4 and the surrounding intronic region of *TMEM38B* was previously identified in Bedouin populations from Israel and Saudi Arabia, we focused on this gene for P1. Nested primers designed to separately detect the specific products from the normal and mutant *TMEM38B* alleles were utilized for PCR amplification, with the expected fragment from the normal allele being detected only in the control sample (Fig 1B). Primers targeting exon 4 failed to amplify P1 gDNA. However, primers flanking the previously reported deletion generated a single 821 bp fragment, consistent with homozygosity for the Bedouin founder mutation, and verified by Sanger sequencing as *TMEM38B* g.32476_53457delins ATTAAGGTATA (Fig 1B).

Proband 2 (P2), a 27-year old American male of English, Scottish and German descent, is the first of two affected children born to nonconsanguineous parents (Fig 1C). His moderately severe OI was diagnosed using the combination of NGS OI gene panels and *TMEM38B* copy number analysis. The initial screening of proband genomic DNA identified an apparently homozygous *TMEM38B* mutation (c.63dupT), which directly introduces a premature termination codon (PTC) in exon 1 (p.D22X). However, only the mother could be confirmed as a carrier for this mutation, while both alleles appeared normal at this site in his father (Fig 1D), suggesting the presence of a paternal *TMEM38B* deletion encompassing exon 1. Subsequent copy number analysis and hemi-nested PCR was used to identify in P2’s father the same deletion previously reported in an Albanian proband, extending from approximately 35 Kb upstream of the start codon to intron 2 of *TMEM38B* [22]. Both affected siblings and the father were confirmed to harbour this mutation, GRCh38 chr9:g.105,682,311_105,716,202del (Fig 1E).

Proband 3 (P3) is a 13-year old male born to consanguineous Pakistani parents residing in Britain (Fig 1F). Following his clinical diagnosis of moderately severe OI, molecular screening using an NGS OI sequencing panel identified the proband as homozygous for a *TMEM38B* c.507G>A point mutation, predicted to introduce a PTC in exon 4 (p.W169X) (Fig 1G), and identical to that recently reported by Lv, *et al* [23]. A complete clinical description of Proband 2 and Proband 3’s phenotype, bone structural and histomorphometric parameters will be published elsewhere.

### The *TMEM38B* mutations result in functional null alleles

**Fig 2:**
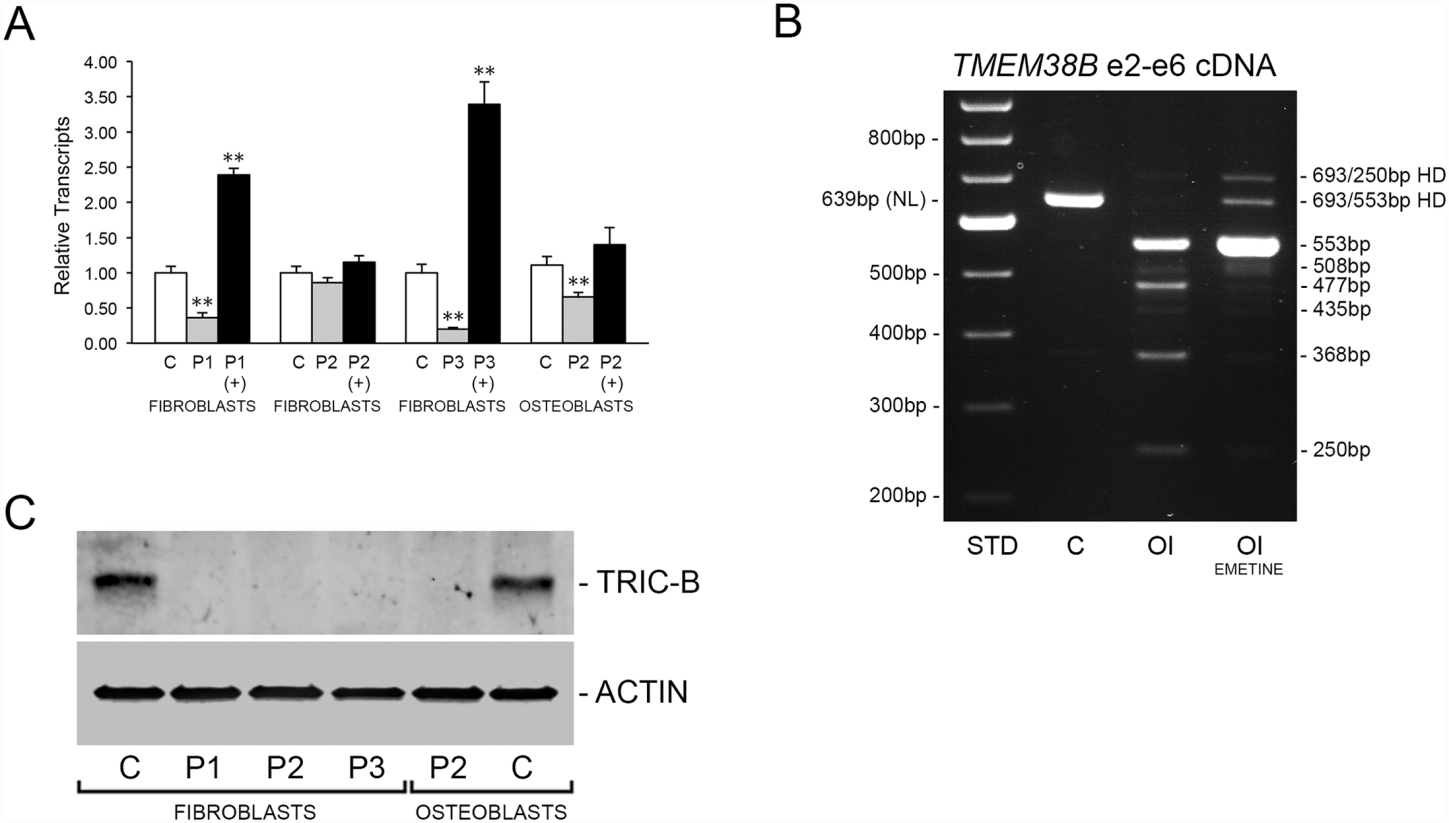
The *TMEM38B* mutations result in null alleles. **(A)** Real-time RT-PCR analysis of *TMEM38B* transcripts from control (C) and proband (P1, P2 and P3) fibroblasts and osteoblasts. Rescue of transcripts by treatment of cells with emetine (+) indicates inhibition of nonsense-mediated decay due to the presence of premature termination codons (PTCs). **, p < 0.01. **(B)** RT-PCR analysis of *TMEM38B* mRNA from control (C) and P1 (OI) fibroblasts in the absence or presence emetine. Several unique amplification products were observed for P1 samples, including multiple splice variants and heteroduplexes (HD), as detailed in S1 Fig. **(C)** Western analysis of control (C) and proband (P1, P2 and P3) fibroblast and osteoblast lysates indicates absence of TRIC-B protein in proband cells.

To determine whether the molecular defects result in generation of abnormal protein products or null alleles, we characterized *TMEM38B* transcripts and TRIC-B protein produced by proband fibroblasts (P1-3) and osteoblasts (P2). By quantitative RT-PCR we found that *TMEM38B* transcripts from Proband 1 cells were reduced to approximately 36 ± 3% of normal control (Fig 2A). P1 *TMEM38B* transcript level was rescued by treatment of cells with emetine, suggesting nonsense-mediated decay (NMD) of transcripts containing premature termination codons (PTCs) (Fig 2A). In agreement, RT-PCR analysis of proband total RNA generated multiple fragments, rather than the normal 639 bp fragment (Fig 2B). Five alternatively spliced transcripts were identified, four of which contain PTCs and one in-frame transcript that was expressed at approximately 8% the level of *TMEM38B* transcripts from control cells (S1A and S1B Fig).

Proband 2 *TMEM38B* transcripts were 86 ± 7% and 66 ± 6% of normal controls in untreated fibroblasts and osteoblasts, respectively (Fig 2A). Since the real-time RT-PCR assay used to quantitate *TMEM38B* expression spans exons 1 and 2, the transcripts detected in P2 cells must originate from the maternal allele, which appears to be capable of significant compensation despite the presence of a PTC. Proband 3 fibroblasts, similar to P1, demonstrated severely reduced transcripts (19 ± 2%) compared to normal control cells, which could be rescued by emetine treatment (Fig 2A).

Although residual *TMEM38B* transcripts were present in proband cells, total absence of TRIC-B protein was demonstrated in all proband fibroblast and osteoblast lysates (Fig 2C). In normal control fibroblasts and osteoblasts *TMEM38A* transcripts were detected at 2–4% of the levels of *TMEM38B* and occurred at equivalent levels in proband cells (S2A Fig), suggesting that TRIC-B is the primary TRIC isoform in these cells. These results establish that the *TMEM38B* mutations identified in our three probands result in functionally null alleles, and its absence is not compensated by increased TRIC-A expression in human fibroblasts and osteoblasts.

### TRIC-B deficiency alters intracellular calcium levels and flux

**Fig 3:**
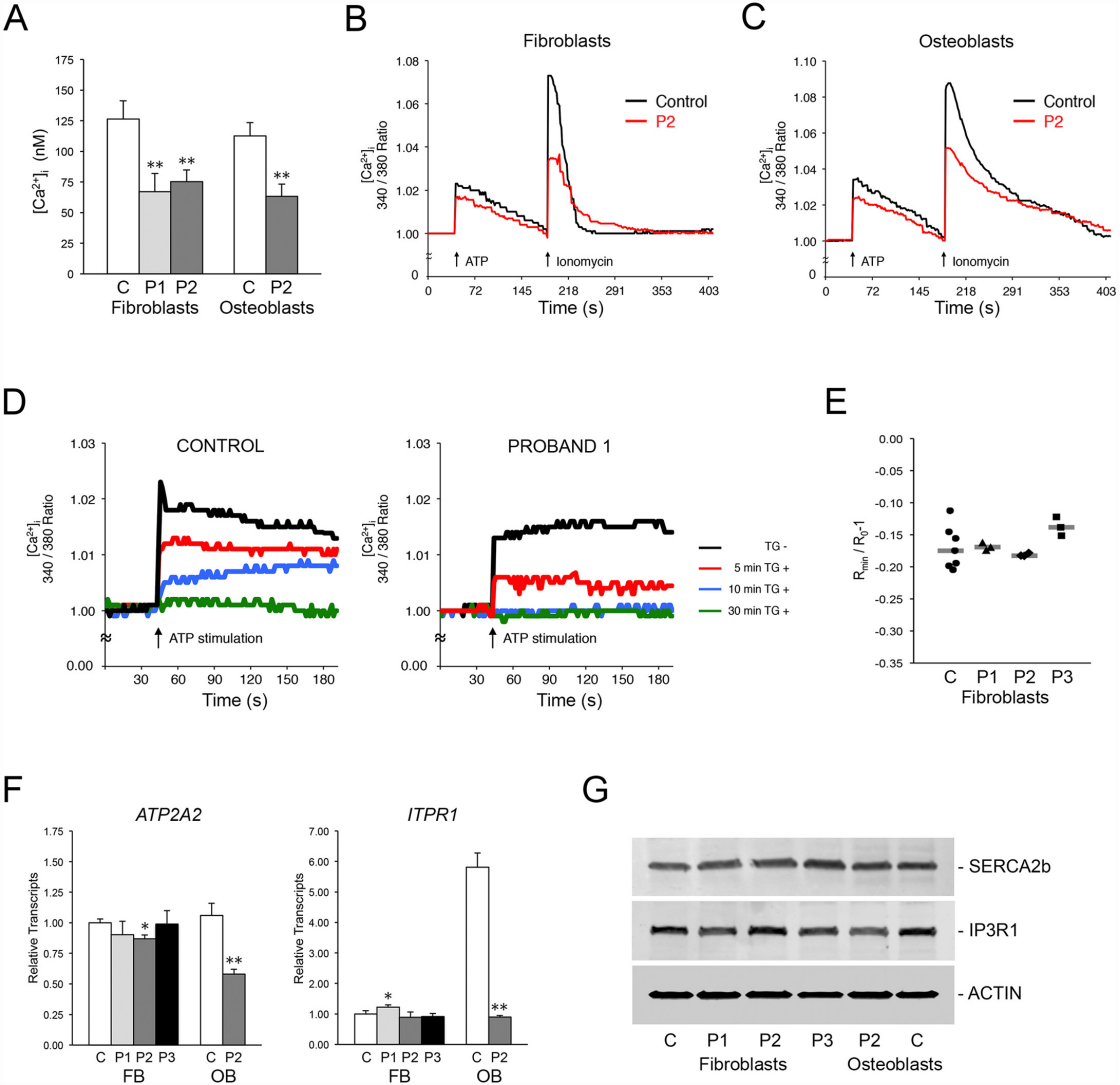
Absence of TRIC-B dysregulates ER Ca^2+^ homeostasis. **(A)** Basal levels of [Ca^2+^]_i_ in control (C) and proband fibroblasts (P1, P2) and osteoblasts (P2). **(B)** Decreased Ca^2+^ mobilization in TRIC-B deficient fibroblasts (red lines). ATP- and Ionomycin-stimulated Ca^2+^ release, as well as the return to baseline levels, are decreased in proband (P2) cells. **(C)** Decreased Ca^2+^ mobilization in TRIC-B deficient osteoblasts (red lines). **(D)** The intracellular Ca^2+^ stores available for IP_3_R-mediated release are more rapidly depleted in Proband 1 fibroblasts (right) versus normal control cells (left). ATP-stimulated Ca^2+^ release is abrogated within 10 minutes in proband cells compared to 30 minutes in control cells following inhibition of SERCA channels with thapsigargin (TG). **(E)** Measurement of ER luminal Ca^2+^ using ER-localized Ca^2+^ indicator. Each point represents the average of one measurement containing 1–7 cells. The difference between the steady-state and Ca^2+^-depleted FRET signal emitted by the D1ER chameleon class Ca^2+^ sensor was equivalent in normal control and proband cells. **(F)** Quantitative RT-PCR in control (C) and proband (P1, P2, P3) cells. There is no significant difference in expression levels of SERCA2 (*ATP2A2*) and IP3R1 (*ITPR1*) in fibroblasts, but transcripts are significantly reduced in proband (P2) osteoblasts. **(G)** Immunoblots of control (C) and proband (P1, P2, P3) cell lysates demonstrate equivalent levels of SERCA2b and IP3R1 Ca^2+^ channels. *, p < 0.05; **, p < 0.01.

In non-excitatory cells, ER Ca^2+^ homeostasis is maintained by SERCA2b, responsible for Ca^2+^ uptake, and IP_3_R, which releases Ca^2+^ stores into the cytoplasm. To verify a role for TRIC-B in ER calcium mobilization, we used a cytoplasmic UV-excitable Ca^2+^ indicator to analyze calcium release from the ER in normal and proband fibroblasts and osteoblasts. In Ca^2+^-free media under steady-state conditions, cytoplasmic [Ca^2+^]_i_ was decreased approximately 40–50% in proband versus normal control cells (Fig 3A), implying a defect in store-operated calcium entry into cells. When IP_3_R-mediated release of Ca^2+^ stores was stimulated with ATP, release of Ca^2+^ from the ER was reduced in proband cells (Fig 3B and 3C). Furthermore, ionomycin-induced Ca^2+^ release from all intracellular stores was also reduced in proband fibroblasts and osteoblasts. The Fura-2 Ca^2+^ indicator signal in proband cells also required a longer period of time to return to baseline than in control cells (Figs 3B, 3C and S3). We further confirmed that Ca^2+^ release from the ER was defective in the absence of TRIC-B by inhibiting Ca^2+^ uptake with thapsigargin. ER Ca^2+^ release was depleted in P1 fibroblasts within 10 minutes of inhibition versus 30 minutes in control cells (Fig 3D).

To determine whether the decreased Ca^2+^ flux in TRIC-B deficient cells was associated with abnormal [Ca^2+^] steady state levels within the ER, we measured free ER [Ca^2+^] using the targeted ratiometric calcium sensor D1ER [25,26]. Interestingly, there was no measurable change in ER luminal free [Ca^2+^] in proband or normal control cells following ATP treatment, suggesting that the amount of stored Ca^2+^ needed to elevate cytoplasmic Ca^2+^ is minor compared to total Ca^2+^ available in the ER. Furthermore, no significant differences in the baseline (before ATP treatment) and depleted (after ionomycin treatment) ER Ca^2+^ signals were observed between normal control and proband cells (Fig 3E). This indicates that TRIC-B deficiency does not result in an altered steady state luminal ER free [Ca^2+^] as, for example, a result of the abnormal [Ca^2+^] flux. Luminal ER free [Ca^2+^] is neither decreased nor increased.

Although ER Ca^2+^ homeostasis was dysregulated in proband cells, transcript levels for *ATP2A2* (ATPase Ca^2+^ transporting cardiac muscle slow twitch 2, SERCA2) and *ITPR1* (IP_3_R1) were normal in proband fibroblasts (Fig 3F). In contrast, in P2 osteoblasts both *ATP2A2* and *ITPR1* expression were decreased to 55 ± 4% and 16 ± 5%, respectively, of normal control osteoblasts. However, no consistent significant difference in SERCA2b or IP_3_R1 protein was detected in proband cells compared to control (Fig 3G). Expression of *RYR1*, encoding the ryanodine receptor for Ca^2+^ release in excitatory cells, was found to be 0.2–1.0% the level of *ITPR1* expression in normal control and proband fibroblasts and osteoblasts (S2B Fig).

### *TMEM38B*-null cells display ER stress

**Fig 4:**
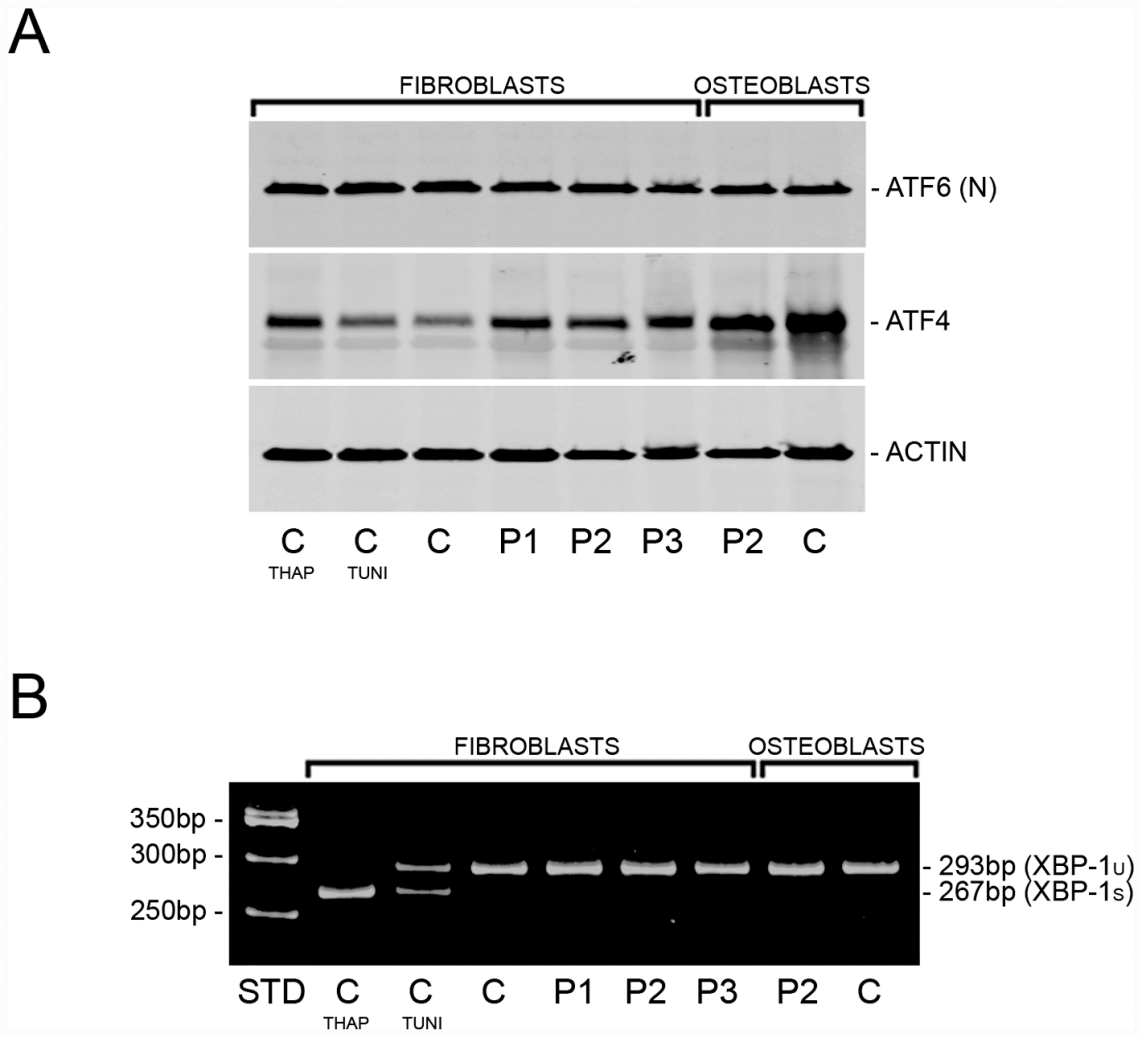
TRIC-B deficiency activates ER stress. **(A)** Immunoblots of steady-state cell lysates demonstrate equivalent levels of ATF6 (N, nuclear form) protein in proband (P1, P2, P3) versus normal control cells. Increased ATF4 protein in proband cells is consistent with activation of the PERK pathway of the UPR, and is similar to the increase in normal control fibroblasts treated with the SERCA inhibitor thapsigargin (THAP), but in contrast to normal control cells treated with N-glycosylation inhibitor tunicamycin (TUNI). **(B)** RT-PCR amplification of *XBP1* mRNA from normal control and proband (P1, P2, P3) cells. There is no difference in the ratio of spliced (XBP-1s) to unspliced (XBP-1u) transcripts between control and proband cells, while partial and complete splicing are detected in normal control fibroblasts treated with tunicamycin (TUNI) and thapsigargin (THAP), respectively.

**Fig 5:**
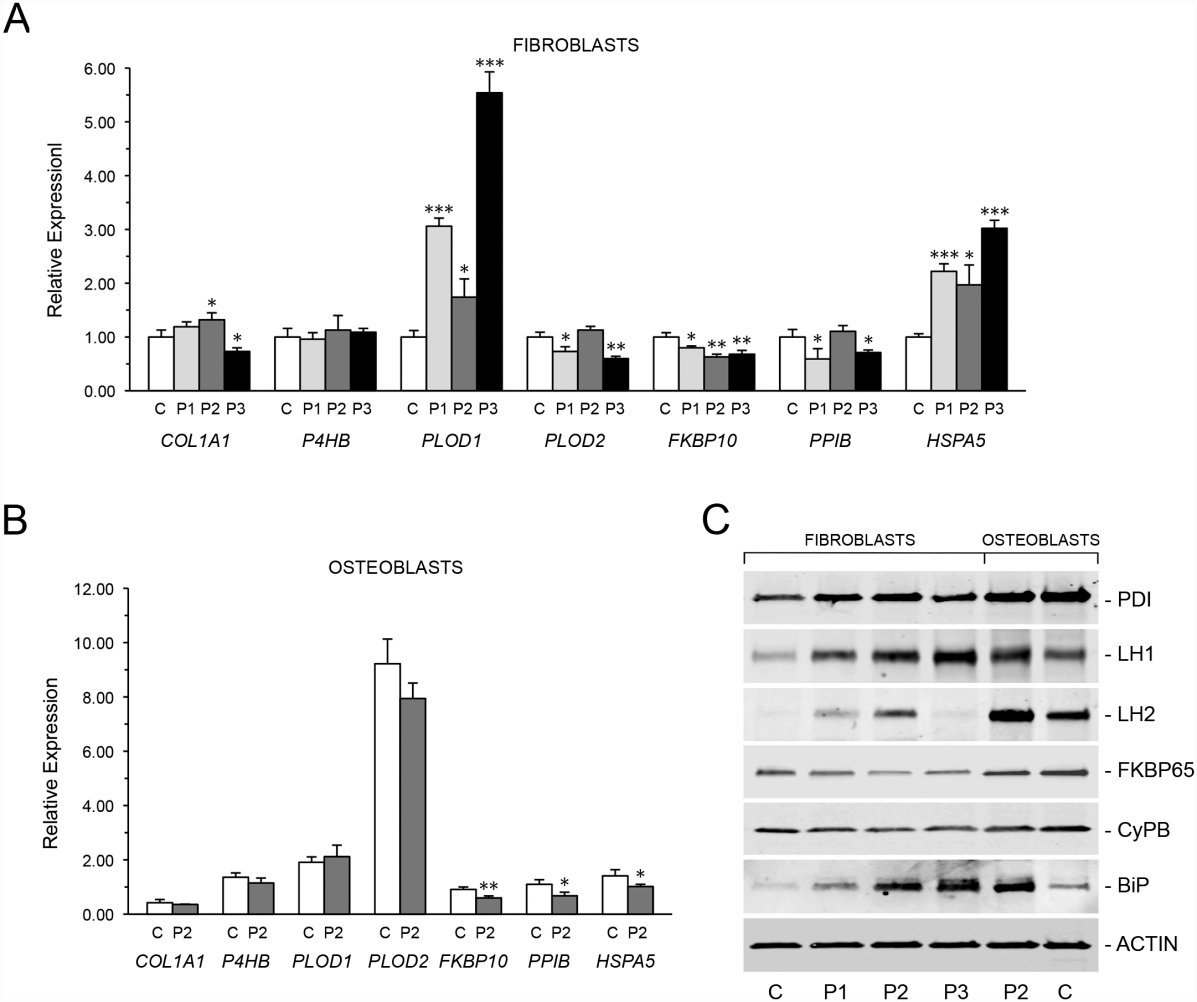
Absence of TRIC-B alters expression of collagen-interacting proteins. **(A)** Quantitative RT-PCR of fibroblast transcripts encoding collagen, modifying enzymes and chaperones. Proband cells (P1, P2, and P3) demonstrate decreased expression of *COL1A1* and *FKBP10* (FKBP65), and increased expression of *PLOD1* (LH1) and *HSPA5* (BiP/GRP78) versus control fibroblasts (C). **(B)** Decreased expression of *PLOD2* (LH2), *FKBP10* (FKBP65) and *PPIB* (CyPB) in P2 osteoblasts versus control osteoblasts (C). **(C)** Immunoblots for quantitation of steady-state protein levels of collagen modifying enzymes and chaperones in proband and control cells. Proband fibroblasts show increases in PDI, LH1, LH2 and BiP protein levels. CyPB and FKBP65, both collagen-interacting isomerases, are consistently decreased in proband fibroblasts and osteoblasts. *, p < 0.05; **, p < 0.01; ***, p < 0.001.

Inhibition of the ER calcium pump (SERCA) is known to trigger an ER stress response in various cell types. We examined the three main signaling pathways activated in the unfolded protein response (UPR) [27], and observed a stress response in TRIC-B deficient cells upon stimulation of procollagen synthesis with ascorbate (Fig 4). Activating transcription factor 4 (ATF4) protein was increased 66–122% in proband fibroblasts versus normal control cells (Fig 4A), suggesting that the PERK-dependent pathway of the UPR is likely activated in these cells. Furthermore, ATF4 is known to interact with the *HSPA5* (BiP/GRP78, immunoglobulin binding protein/78 kDa glucose-regulated protein) promoter [28], consistent with our findings that *HSPA5* transcripts and BiP protein were increased 45–122% and 145–332%, respectively, in proband fibroblasts, and associated with a 250% increase in BiP in P2 osteoblasts compared to normal control osteoblasts (Fig 5A–5C). In contrast, cleavage of ATF6 by Site 1- (S1P) and Site-2 proteases (S2P) and IRE1-mediated splicing of *XBP1* were both found to be equivalent in proband and normal control cells by western and RT-PCR analyses (Fig 4A and 4B).

### TRIC-B deficiency affects collagen-modifying enzymes

We next investigated the effects of TRIC-B deficiency on collagen-modifying enzymes in proband cells (Fig 5A–5C). Expression studies of proteins previously demonstrated to be calcium-dependent revealed that FK506 binding protein 10 (*FKBP10*) transcripts were reduced 31–46% versus normal controls, and its protein product FKBP65, which is stabilized by Ca^2+^ and is required for collagen telopeptidyl hydroxylation and crosslinking, was decreased approximately one-half in proband fibroblasts and osteoblasts versus matched normal control cultures. We found additional alterations in the expression of genes that have not previously been demonstrated to require calcium for stability or function. Procollagen-lysine, 2-oxoglutarate 5-dioxygenase 1 (*PLOD1*) transcripts were increased 50–360% and its protein product, lysyl hydroxylase 1 (LH1), was increased 123–180% in proband fibroblasts and 70% in osteoblasts, compared to matched normal control cells. *PLOD2* transcripts were decreased 30–40% in P1 and P3 fibroblasts, and 15% in P2 osteoblasts, but LH2 protein was found to be increased 25–600% in proband fibroblasts and 100% in osteoblasts versus matched normal control cells. Although no consistent changes in expression were observed for protein disulfide isomerase/prolyl 4-hydroxylase subunit beta (PDI/*P4HB*) or Cyclophilin B (CyPB/*PPIB*) transcripts, we found consistent alterations in PDI and CyPB protein levels. Immunoblots demonstrated that proband cell lysates contained 48–124% more PDI protein versus matched normal control cells. In contrast, CyPB protein was decreased 17–50% versus matched controls.

### TRIC-B deficiency alters collagen post-translational modification and assembly

**Fig 6:**
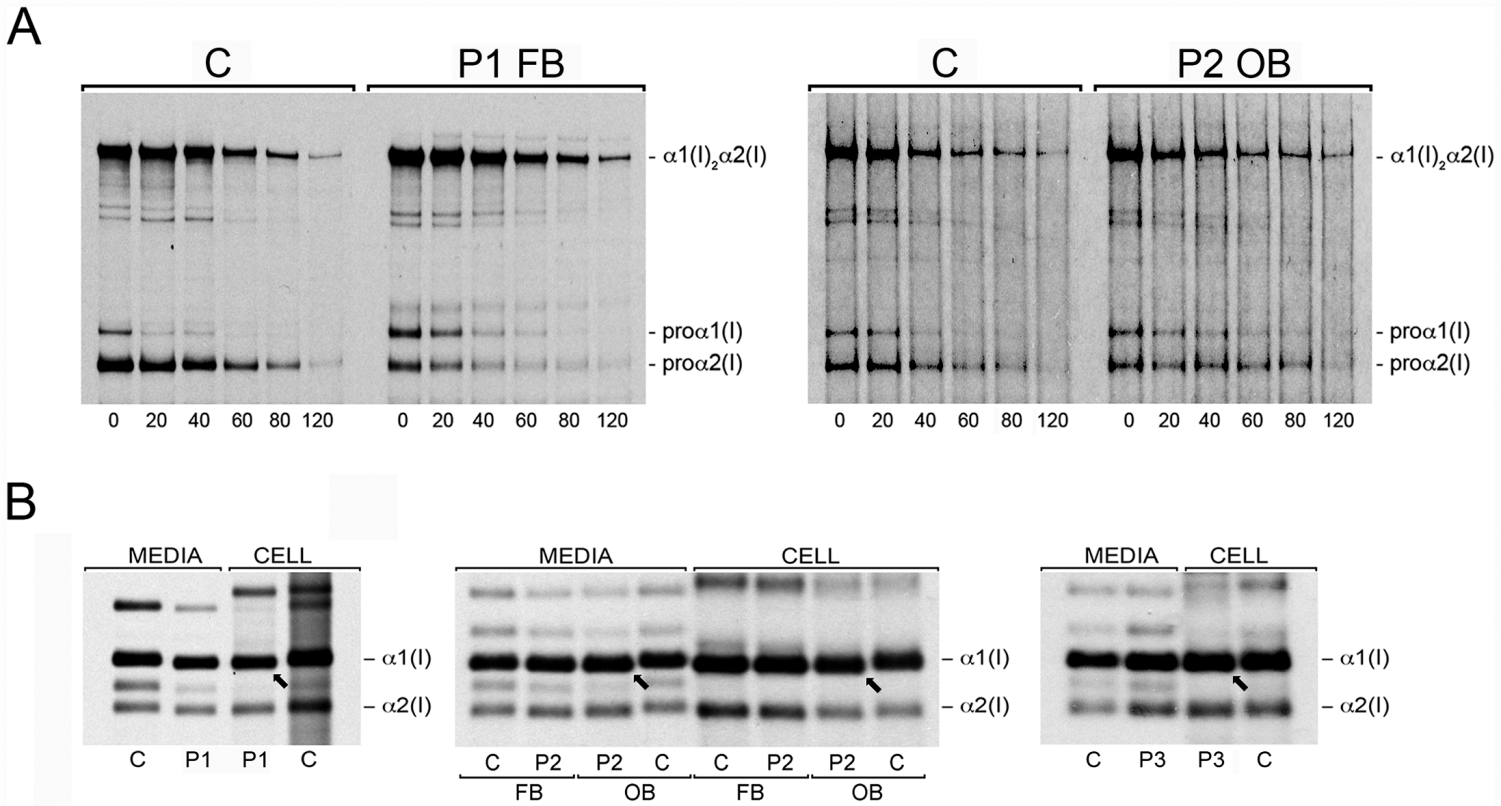
Disruption of ER [Ca^2+^] homeostasis dysregulates type I collagen synthesis. **(A)** Intracellular chain association assay to follow assembly of monomeric pro-α1(I) and pro-α2(I) chains into heterotrimeric procollagen molecules, α1(I)_2_α2(I), at 20 minute intervals of a pulse-chase analysis. Unincorporated pro-α1(I) chains are present in proband cells approximately 20 minutes longer in P1 fibroblasts (P1 FB) and P2 (P2 OB) osteoblasts versus normal control fibroblasts and osteoblasts (C). **(B)** Steady-state cell layer (cell) and secreted (media) collagen from normal control (C) and proband fibroblasts (P1-P3, FB) and osteoblasts (P2, OB) analyzed by SDS-Urea PAGE. Both the α1(I) and α2(I) chains of type I collagen from proband cells demonstrate faster electrophoretic migration than normal control type I collagen alpha chains.

Given the alterations in collagen-interacting protein levels in proband cells, we examined type I collagen modification and folding. PDI (*P4HB*) catalyzes the formation and rearrangement of disulfide bonds, which is a critical step in the initial association and folding of procollagen alpha chains. Although we found that *P4HB* (PDI) expression was normal (Fig 5A and 5B), and PDI protein levels were moderately increased (Fig 5C), there was an approximately 20 minute delay in procollagen chain assembly into heterotrimeric molecules in proband fibroblasts and osteoblasts (Fig 6A). Furthermore, following alpha chain association, CyPB facilitates collagen folding into a protease-resistant triple helical conformation by catalyzing the *cis-trans* isomerization of collagen peptidyl-prolyl bonds. In direct intracellular collagen folding assays, the rate of accumulation of intracellular protease-resistant type I collagen heterotrimers in TRIC-B-deficient cells was equivalent to control cells, indicating that the peptidyl-prolyl isomerase function of CyPB is sufficient for normal collagen folding (S4 Fig).

In classical OI, collagen structural defects delay folding of the helix and increase the duration of exposure to ER-resident modifying enzymes, including LH1. In contrast, LH1 deficiency in Type VI Ehlers-Danlos (EDS VI) results in decreased modification of the collagen alpha chains. These alterations in collagen post-translational modification can be detected as delayed or increased electrophoretic migration, respectively, of the collagen alpha chains. Surprisingly, despite consistent increases in transcript and protein levels of *PLOD1*/LH1 in TRIC-B-deficient cells (Fig 5A–5C), helical lysine hydroxylation was reduced by approximately 20–30% in proband fibroblast type I collagen (Table 1). By comparison, fibroblasts from a proband with EDS VI synthesize type I collagen with an 86% reduction in hydroxylysine content (Table 1). Reduced hydroxylysine content is also consistent with the more rapid electrophoretic migration of proband type I collagen alpha chains on SDS-Urea-PAGE (Fig 6B). Type I collagen synthesized by proband osteoblasts has the same increased electrophoretic mobility as proband fibroblast collagen, but shows a lesser extent of reduction in total hydroxylysine content compared to published data on differentiated human bone marrow stromal cells (Table 1) [29]. The decreased hydroxylation of collagen lysyl residues synthesized by TRIC-B deficient cells demonstrates impaired lysyl hydroxylase function, despite increased transcript and protein levels.

**Table 1:** Type I procollagen post-translational modification.

	NL Control FB	Proband 1 FB	Proband 2 FB	Proband 3 FB	EDS VI FB	Proband 2 OB	NL Control OB
% Total Hyl	22.7 ± 2.8	15.9	16.5	18.8	3.2	21.1	23.2 ± 2.5*
% Total Hyp	44.6 ± 1.3	47.0	43.1	36.4	53.0	41.5	44.5 ± 0.7*
% α1(I) P986 3-Hyp	95.5 ± 1.5	89	83	88		91	95
% α1(I) N-telo Hyl	17	26	32	37		26	27
% α2(I) N-telo Hyl	69	84	100	100		100	100
% α1(I) C-telo Hyl	62.3 ± 3.7	86	64	85		100	100

* Values for fully differentiated human BMSC cultures previously reported by Uzawa, *et al* [29].

Interestingly, collagen telopeptidyl hydroxylation, catalyzed by FKBP65-dependent LH2 (*PLOD2*), was increased despite the reduction in *FKBP10* transcripts and FKBP65 protein to the level of type XI OI carriers (OMIM #610968, Fig 5A–5C, Table 1) [30]. Also, modification of proline residues in proband type I collagen catalyzed by prolyl 4-hydroxylase was normal, while collagen prolyl 3-hydroxylation was comparable to the level found in carriers of types VII and VIII OI (OMIM #610682, #610915, Table 1) [31,32].

### Type I collagen synthesized by TRIC-B deficient cells has an abnormal conformation

**Fig 7:**
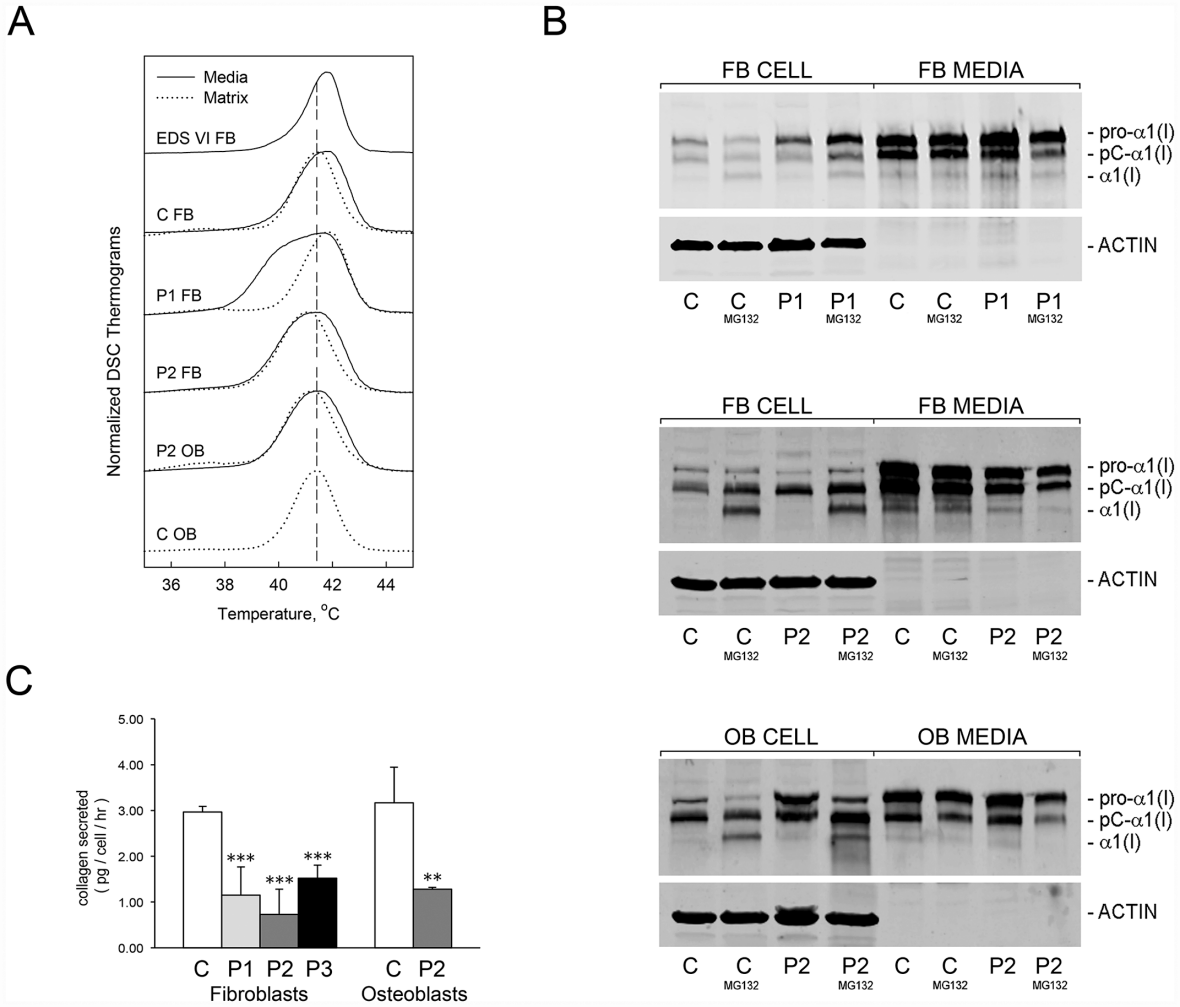
Collagen conformation and secretion are altered by abnormal ER [Ca^2+^]. **(A)** Differential scanning calorimetry (DSC) analysis reveals no differences in thermal stability (T_m_) of type I collagen secreted by control (C, media) and LH1-deficient (EDS VI, media) fibroblast cultures, but a low-stability fraction in proband (P1, P2, media) secreted collagen. Thermal stability of type I collagen extracted from extracellular matrix deposited in culture by control (C, matrix) and proband (P1, P2, matrix) cells is equivalent. **(B)** Immunoblots of intracellular (cell) and secreted (media) type I collagen from control (C) or proband (P1, P2) fibroblasts (FB) and osteoblasts (OB, P2) cultured in the absence or presence of proteasomal inhibitor (MG132). Retention of type I collagen in proband cells is increased upon treatment. **(C)** Quantitation of total procollagens in 24 hour conditioned media of control (C) and proband cell cultures. Secretion of procollagens is decreased by more than one-half in proband fibroblasts (P1, P2, P3) and osteoblasts (P2). **, p < 0.01; ***, p < 0.001.

Because proband collagen post-translational modification was altered, we sought to characterize its structure and stability. Calorimetric analysis of secreted type I collagen showed a lower stability fraction in proband collagen samples, with reduction in T_m_ ranging from 2°C in P1 to 0.5°C in P2. In P1 collagen, the lower stability fraction comprised 30–40% of total secreted proband collagen (Fig 7A). In contrast, the melting curve of collagen secreted from *PLOD1* (LH1)-deficient fibroblasts (EDS VI) was similar to control collagen, demonstrating that destabilization of collagen is not a consequence of the underhydroxylation of lysyl residues, and raising the probability that it is a function of improper folding.

The low stability collagen synthesized in the absence of TRIC-B is susceptible to intracellular retention and proteasomal degradation. Immunoblots of cell lysates and conditioned media demonstrated increased cell layer type I procollagen in proband fibroblasts (P1, 285 ± 64%; P2, 65 ± 25%) and osteoblasts (P2, 83 ± 24%) versus normal control cells (Fig 7B). Although no significant difference in the total amount of cell layer procollagen was seen in normal control cells treated with MG132, proteasomal inhibition further increased the accumulation of intracellular type I procollagen in proband fibroblasts (P1, 335 ± 78%; P2, 220 ± 90%) and osteoblasts (P2, 355 ± 21%), with a concomitant 40 ± 10% and 57 ± 5% reduction of collagen secreted into the media of proband fibroblasts and osteoblasts, respectively. The disproportionate distribution of collagen between the cell layer and media fraction in proband cultures is consistent with the presence of a misfolded collagen species that is predominantly retained and degraded by ER-associated degradation (ERAD).

A fraction of the misfolded collagen eludes intracellular degradation and is secreted into the extracellular space, although the proportion varies among probands and between osteoblasts and fibroblasts. Pulse-chase analysis of procollagen secretion demonstrated that pericellular processing in P1 fibroblast cultures was delayed, especially at the amino end of the pro-α1(I) chain (S5 Fig). Since the type I procollagen amino propeptidase is a conformation-sensitive enzyme, these results further suggest that the conformation of procollagen secreted by P1 fibroblasts is abnormal. The lower thermal stability, increased intracellular proteasomal degradation and resistance to processing by endogenous enzymes support the presence of a misfolded collagen species in TRIC-B-deficient fibroblasts, some of which is secreted.

### Decreased synthesis, secretion and deposition of abnormal Type I collagen causes extracellular matrix insufficiency

Given the abnormal collagen post-translational modification and conformation, we investigated the effects of TRIC-B deficiency on collagen secretion and incorporation into extracellular matrix deposited in culture. The intracellular retention and degradation results in a 50–75% (p < 0.01) decrease in collagen secretion from proband versus control fibroblasts and osteoblasts, as quantitated in 24-hour conditioned media (Fig 7C). The decreased amount of procollagen secreted by proband cells did not reflect a delay traversing the secretory pathway, as the secretion rate was normal. Rather, total type I procollagen synthesized by P1 fibroblasts was decreased by 38 ± 7% (p < 0.01) compared to normal control cells (S6 Fig).

The low stability collagen species identified by DSC in the media of P1 fibroblast cultures was not incorporated into extracellular matrix deposited in culture, which contained only collagen with normal thermal stability (Fig 7A), and was associated with a 35 ± 5% reduction of crosslinked collagen in matrix (S7 Fig). Thus, reduced secretion of total procollagen, exacerbated by the presence of misfolded collagen species that are resistant to incorporation into extracellular matrix, together contribute to matrix insufficiency.

## Discussion

*TMEM38B*, encoding the integral ER membrane protein TRIC-B, was first identified as a novel gene causing autosomal recessive OI among consanguineous Bedouin kindreds from Saudi Arabia and Israel, who harboured a homozygous exon 4 deletion mutation [20,21]. A second homozygous deletion mutation, encompassing the first two exons of *TMEM38B*, was identified in an Albanian proband [22]. And two point mutations, affecting either the intron 3 splice acceptor site or an exon 4 nonsense mutation, were reported in Chinese Han probands. [23]. The three cases reported in this investigation involved three of the four previously reported *TMEM38B* mutant alleles, and one additional independent *TMEM38B* mutation in a family of English/Scottish background, all resulting in total absence of TRIC-B protein due to nonsense mutations. Patients with type XIV OI have low bone volume associated with low bone turnover and relatively normal bone matrix mineralization, features that set this OI type apart from the high bone turnover and increased mineralization found in classical OI. Here, we utilized the *TMEM38B*-null primary fibroblasts and osteoblasts from our probands to characterize the mechanism by which absence of TRIC-B causes moderately severe OI.

This report also presents the first demonstration in humans of abnormal ER Ca^2+^ homeostasis due to the absence of TRIC-B. In proband primary fibroblasts and osteoblasts we found that both cytoplasmic [Ca^2+^] and IP_3_R-mediated Ca^2+^-flux were decreased in Ca^2+^-free media, genetically validating a role for TRIC-B in maintaining intracellular Ca^2+^ levels [24]. Ionomycin-induced Ca^2+^ flux from the ER into the cytoplasm was weaker in proband cells. Deficient Ca^2+^ release from the ER was also found after ER calcium pumps were irreversibly inhibited with thapsigargin; the calcium available for release from the ER was depleted more rapidly from TRIC-B deficient than from control cells. The decreased cytoplasmic Ca^2+^ in *TMEM38B*-deficient cells suggested Ca^2+^ transport processes at the cell membrane may also be altered. Calcium entry into the cell by a process of “store-operated calcium entry” (SOCE) may be secondarily defective in *TMEM38B*-deficient cells, possibly due to defective function of the ER Ca^2+^ sensor STIM protein [33]. Interestingly, the steady state ER Ca^2+^ measurement in live cells with the direct ER sensor D1ER was neither increased nor decreased compared to control. This data implies that cells with chronic TRIC-B deficiency make compensatory adjustments in ER calcium uptake, such as upregulation of stimulated Ca^2+^ release channel function, which may also underlie the rapid Ca^2+^ depletion seen with the cytoplasmic Fura-2 probe on ATP stimulation. Taken together, the type XIV OI ER and flux data support a role for TRIC-B channels when calcium release channels are closed, presumably by assuring a slow return to normal ER potassium homeostasis. Deficiency of TRIC-B likely slows ER Ca^2+^ recovery more than it affects ER Ca^2+^ steady state.

Studies of TRIC-A and TRIC-B function in murine cells, cardiomyocytes or type II alveolar cells have led to two proposals for the TRIC-B contribution to ER Ca^2+^ homeostasis. In one model, Takeshima and colleagues proposed that TRIC-A channels selectively provide K^+^ countercurrents in support of RyRs, while TRIC-B serves to support IP_3_R-mediated Ca^2+^ release [34]. Murine *Tricb* ^-/-^ type II alveolar cells demonstrated decreased intracellular resting Ca^2+^ levels and weakened ATP-induced IP_3_R-mediated Ca^2+^ release, but increased Ca^2+^ responses to ionomycin in comparison to wild-type cells when the calcium indicator is loading in the presence of Ca^2+^ [35]. They proposed that a state of ER Ca^2+^ overload exists when TRIC-B is absent. In a second model, Fill and colleagues proposed that the major Ca^2+^/K^+^ countercurrent occurs directly through RyR and IP_3_Rs, rather than through TRIC channels [36]. This group also observed no change in ER Ca^2+^ activity when TRIC function was acutely limited [37]. Instead, TRIC channels would function to maintain the K^+^ steady state when Ca^2+^ release channels are not gating [37]. Hence, if TRIC-B is a calcium sensor that detects a differential [Ca^2+^]_i_ between the cytosol and lumen [38], then ER calcium replenishment would be defective in the absence of TRIC-B. Our experiments detected no genetic support for the ER Ca^2+^ overload model in osteoblasts, and are rather consistent with a role for TRIC-B in modulating the kinetics of Ca^2+^ flux. Although the osteoblast cell line MC3T3-E1 has been reported to have functional IP_3_R and RyR receptors for Ca^2+^ release [39], we found negligible expression of RyR, and minimal TRIC-A expression (2–5% of TRIC-B) in proband cells. In murine lung cells, TRIC-A protein is several-fold lower than TRIC-B [35]. Thus, the difference between mouse alveolar type II cells and human fibroblasts/ osteoblasts may represent cell- or species-specific redundancy of channels for Ca^2+^ flux.

Inhibition of the ER calcium SERCA-pump elicits a stress response in mammalian cells, characterized by dissociation of BiP from PERK, IRE1 and ATF6 [40–42]. We found that the abnormal Ca^2+^ flux in TRIC-B deficient cells increased ATF4, consistent with PERK activation, but not IRE1 and ATF6. The PERK branch of the UPR has been proposed to mediate cellular responses to calcium concentration, and may function as an ER Ca^2+^ sensor [43].

**Fig 8:**
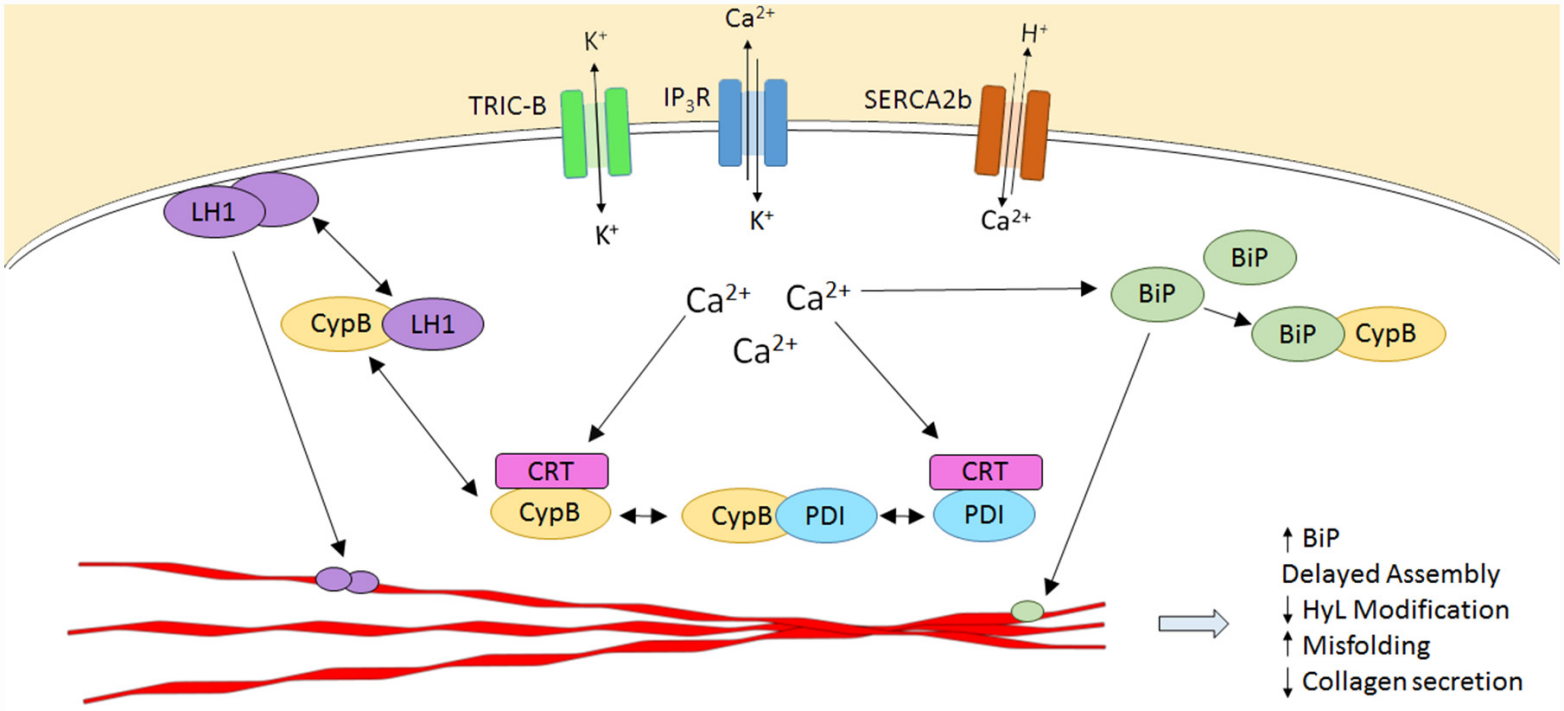
Calcium flux regulates collagen post-translational interactions within the ER. Secreted proteins such as collagen are synthesized within the ER, which contains the major intracellular Ca^2+^ store. In response to extracellular stimuli, Ca^2+^ is released from the ER lumen into the cytoplasm via IP_3_ and ryanodine receptors. Cytoplasmic Ca^2+^ that does not bind signaling proteins involved in gene expression is transported back into the ER via SERCA pumps. The continuous fluctuations in free [Ca^2+^] to and from the ER are indirectly regulated by TRIC channel activity, which mediates transmembrane K^+^ flux to maintain electroneutrality across the ER membrane. In fibroblasts and osteoblasts, ER-resident Ca^2+^-binding chaperones, including BiP, CyPB, PDI and calreticulin (CRT), directly interact with collagen alpha chains (red polypeptides) to facilitate or catalyze specific modifications required for assembly and folding. In the absence of TRIC-mediated K^+^ flux, Ca2^+^-dependent “cycling” of these chaperones between inactive, sequestered forms and active, substrate-interacting forms, is dysregulated. Thus, absence of TRIC-B alters ER luminal [Ca^2+^], affecting synthesis and secretion of collagen, as well as interactions of collagen-specific chaperones with each other and with their substrate, resulting in abnormal collagen assembly and post-translational modification. BiP, GRP78; CyPB, cyclophilin B; CRT, calreticulin; IP3R, inositol triphosphate receptor; LH1, lysyl hydroxylase 1; PDI, protein disulfide isomerase; SERCA2b, sarcoplasmic-endoplasmic reticulum Ca^2+^-ATPase isoform 2b; TRIC-B, trimeric intracellular cation channel subtype B.

Why do null mutations for *TMEM38B* cause osteogenesis imperfecta, which is a collagen-related condition? We show here that disrupted ER-Ca^2+^ flux kinetics in *TMEM38B*-null cells is associated with altered expression and activity of multiple collagen modifying enzymes and chaperones, resulting in abnormal post-translational modification and folding of type I collagen (Fig 8). We found abnormal levels and/or functioning of collagen-interacting proteins BiP, PDI and CyPB in patient cells. The delay in trimeric procollagen chain assembly in proband cells suggests that the ability of PDI to catalyze disulfide formation may be compromised, despite the increased quantities of PDI protein in proband cells. Because of slow Ca^2+^ flux kinetics, PDI may be sequestered by binding to calreticulin [14,44], and have reduced availability for collagen folding. CyPB, a member of the procollagen 3-hydroxylation complex in the ER, was also consistently decreased in proband cells, although not below the level expected of an unaffected heterozygous carrier of a *PPIB* defect [30]. Proband type I collagen shows moderately decreased 3Hyp levels, similar to heterozygous carriers of null mutations P3H1 and CRTAP (Table 1) [31,32,45], implying some impairment in complex function. In addition, CyPB’s role as a binding partner for multiple ER chaperones and foldases may be impaired in *TMEM38B*-null cells. CyPB binds both BiP and PDI, as well as a number of other ER partners, through a lysine-rich site distinct from the isomerase catalytic site inhibited by cyclosporin A (CsA). The partner proteins bind to CyPB through an N-terminal acidic motif that has also been suggested to be a calcium-binding site [46]. Interactions between CyPB and its partners have been speculated to concentrate both proteins on shared substrates [46], and disruption of these interactions in an altered ER [Ca^2+^] environment might modulate their joint role. In contrast, we detected no delay in collagen folding, which is dependent on the PPIase function of CyPB [47].

The most striking alteration in collagen modification is the reduction in hydroxylysine content of proband collagen, presumably due to dysfunction of LH1, despite the attempted cellular compensation of increased *PLOD1* transcripts and increased LH1 protein. *In vitro*, only high non-physiologic [Ca^2+^] levels were found to decrease the activity of purified LH1 [48]. *In vivo*, LH1 protein has been shown to interact directly with CyPB and SC65/P3H3 [47,49,50]. Since LH1 dysfunction in *TMEM38B*-null cells cannot be attributed to absence of the PPIase function of CyPB, it raises the speculation that LH1 and CyPB might have an additional Ca^2+^-regulated interaction, similar to that demonstrated for other chaperones. The collagen lysine hydroxylation defect in *TMEM38B*-null cells may be more selective for certain residues in osteoblast than fibroblast collagen. Residue-specific alteration of lysine hydroxylation in fibroblasts and osteoblasts occurs in *Ppib*-null and *Sc65*-null mice [49,50]. Because data on lysine modification of collagen from normal human primary osteoblasts is not available, we compared our proband’s osteoblast collagen to published data from differentiated human bone marrow stromal cells (BMSC) [29], which may vary in modification from collagen synthesized by primary osteoblasts. Regardless, the altered hydroxylysine content of collagen synthesized by *TMEM38B*-null patient cells would reduce the residues available for crosslinking of collagen molecules in matrix, which contributes to matrix stability, bone strength and ductility [51,52].

Interestingly, although the immunophilin FKBP65, which is stabilized by Ca^2+^ and modifies collagen telopeptidyl residues involved in crosslinking by LH2 [53,54], was decreased in proband cells, collagen telopeptidyl hydroxylysine content was moderately increased. This likely reflects slower collagen assembly in the presence of sufficient residual FKBP65 activity.

In summary, our data demonstrate that absence of TRIC-B from fibroblasts and osteoblasts of patients with type XIV OI impairs ER [Ca^2+^] flux kinetics. We identified dysregulation of multiple steps in type I collagen synthesis and modification in proband cells, placing type XIV OI within the collagen-related paradigm of OI. Ca^2+^/K^+^ flux abnormalities can reasonably be expected to affect synthesis and secretion of additional matrix components beyond collagen. Furthermore, Ca^2+^/K^+^ flux abnormalities likely affect bone cell function, since intracellular calcium signaling is known to control osteoblast proliferation and differentiation, as well as osteoclast survival and resorptive activity, through regulation of gene transcription [55–59].

## Materials and Methods

### Human subjects

This study was approved by the NICHD IRB (Protocol #04-CH-0077). Cells from Proband 3 were obtained as part of the diagnostic process. Written, informed consent was obtained from all subjects or their respective guardians.

### Cell culture

Proband and three independent normal control primary fibroblast (FB) cell lines were cultured at 37°C and 5% CO_2_ in DMEM with GlutaMAX (Life Technologies) containing 10% fetal bovine serum and 1% pen-strep. Primary osteoblast (OB) cultures were established from surgical bone chips of normal control and Proband 2. Following digestion of bone chips for 2 h at 37°C with 0.3 units/ml collagenase P in serum-free αMEM, osteoblasts were cultured in αMEM supplemented with 10% fetal bovine serum and 1% pen-strep at 37°C and 8% CO_2_ [60]. For generation of conditioned media, cells were cultured in media containing 0.1% fetal bovine serum and 100 μg/ml ascorbate for 24 h in the presence or absence of 50 μM MG132 (Sigma-Aldrich). For ER stress experiments, cells were cultured in media supplemented with 100 μg/ml ascorbate with or without N-[N-(N-Acetyl-L-leucyl)-L-leucyl]-L-norleucine (ALLN, Sigma-Aldrich) for 8 h.

### Mutation identification

Total RNA was extracted from fibroblast and osteoblast cultures in the presence or absence of emetine (100 μg/ml) using TriReagent (Molecular Research Center) according to the manufacturer’s protocol. Total RNA was treated with DNA-free (Life Technologies) prior to further analysis. Since the majority of cases of OI are caused by dominant mutations in *COL1A1* and *COL1A2*, molecular screening for a causative defect began with these genes. No sequence variants, however, were detected in cDNA obtained from proband fibroblast cultures.

Genomic DNA (gDNA) was prepared from fibroblasts or whole blood using the Puregene DNA extraction kit (Qiagen). Additional molecular screening was performed on proband genomic DNA utilizing Next Generation Sequencing (NGS) covering a panel of genes associated with autosomal dominant and recessive forms of OI (*ALPL*, *BMP1*, *COL1A1*, *COL1A2*, *CRTAP*, *FKBP10*, *IFITM5*, *LEPRE1*, *PLOD2*, *PPIB*, *SERPINF1*, *SERPINH1*, *SP7*, *TMEM38B* and *WNT1*; Connective Tissue Gene Tests).

For Proband 1, fibroblast gDNA (500 ng) was used to amplify the region surrounding the putative deletion inclusive of exon 4 in *TMEM38B* (NG_032971.1), which was previously characterized by Volodarsky *et al* [21]. A fully nested PCR was performed using 15 pmol primers flanking exon 4, which are specific to the normal allele (WT-S, 5’-TGG TGA AAG GGA AGA ATT GC-3’; WT-AS, 5’-GAG AAA CCC ACA GAG AAG GAA A-3’), and primers flanking the deletion (Exon 3s, 5’-CCG CAT GAC CTA GTT TCC CAG GGC TAT TCA-3’; Intron 4as, 5’-CAA AGC AGG ATG AGG TTT GGA CAA CAG ACC-3’), 1% Perfect Match (Agilent Technologies), 3% DMSO, and 1.0 U High Fidelity Platinum Taq polymerase (Life Technologies). After an initial 5 minute denaturation at 94°C, reactions were incubated for 35 cycles of 94°C for 1 min, 51°C for 30 sec and 72°C for 1 min, followed by 7 min at 72°C. PCR products were then sequenced (Macrogen). Genomic DNA was unavailable for further testing of the parents or siblings.

For Proband 2, NGS (Connective Tissue Gene Tests) identified only a single mutant allele, which was verified by Sanger sequencing. Genomic DNA from the proband and his family members was used to amplify the region surrounding exon 1 of TMEM38B by PCR. Reactions contained 15 pmol primers (5’UTRs, 5’-TCT CCT ACT CCT CAC CGC-3’; Intron 1as, 5’-CGA CTT AGA CGG TCC TCG-3’) and 1.25 U Amplitaq Gold (Life Technologies). Following an initial 10 minute denaturation at 95°C, reactions were incubated for 40 cycles of 94°C for 30 sec, 54°C for 30 sec and 72°C for 30 sec, followed by 7 min at 72°C. PCR products were then sequenced (Macrogen). The second mutant allele in Family 2 was detected by high-density targeted (HDT) array, which suggested copy number variation (CNV) in the first two exons of *TMEM38B* for the proband, his sibling and father (Connective Tissue Gene Tests). Based on the molecular findings reported by Rubinato *et al* [22], heminested primers were designed to amplify the region encompassing a 35 Kb region encompassing exons 1 and 2 of *TMEM38B*. PCR amplification, using 15 pmol primers (5’BP-S, 5’-GCA GCC TCA ACC TCC TGG GCT CAG ATG ATT-3’; 5’WT-AS, TAG GCC TGG CTG GTG GCT CAT GCC TGT AAT-3’; 3BP-AS, 5’-GCT TTT GAA ACT GAC CCA CCA GGG CTC TCT-3’) and the same conditions described above, generated 216 bp and 339 bp fragments for the mutant and normal alleles, respectively.

Proband 3 genomic DNA was amplified using 3% DMSO, 1.0 U High Fidelity Platinum Taq polymerase (Life Technologies), and 15 pmol primers encompassing exon 4 of *TMEM38B* (INT3-S, 5’-TTG ACA TGC TGG TGA AAG GGA AGA ATT GCA-3’; INT4-AS, 5’-CAG CTA TAT TCC ACC CAC ATT TAA AGT CCC-3’). An initial 5 minute denaturation at 94°C was followed by 35 cycles of 94°C for 1 min, 60°C for 30 sec and 72°C for 1 min, followed by 7 min at 72°C. Amplification products were then sequenced (Macrogen).

### Analysis of gene expression

Gene transcript levels were quantitated by real-time RT-PCR following reverse-transcription using a High Capacity cDNA Archive Kit and Taqman Assays on Demand (Life Technologies, *TMEM38A*, Hs00225325_m1; *TMEM38B*, Hs00216531_m1; *COL1A1*, Hs00164004_m1; *FKBP10*, Hs01000263_m1; *PLOD1*, Hs00609368_m1; *PLOD2*, Hs01118190_m1; *PLOD3*, Hs01126617_m1; *P4HB/PDI*, Hs00168586_m1; *HSPA5*, Hs99999174_m1; *PPIB*, Hs00168719_m1; *ATP2A/SERCA2*, Hs00544877_m1; *ITPR1*, Hs00181881_m1; *RYR1*, Hs00166991_m1; *GAPDH*, Hs99999905_m1; *ACTB*, Hs99999903_m1; *B2M*, Hs99999907_m1). Relative expression of genes of interest was measured in triplicate in 3 independent cultures against 3 reference genes (*ACTB*, *B2M* and *GAPDH*). To validate gene expression levels in normal control fibroblast cell lines, transcript levels were determined independently for each control cell line and compared to cDNA derived from pooled RNA samples (S8 Fig). Gene expression in proband fibroblasts was then quantified relative to pooled normal control fibroblast values. Gene transcripts in proband osteoblasts are expressed relative to a single normal control primary osteoblast cell line.

Identification of *TMEM38B* splice variants in P1 mRNAwas performed as follows: DNase-treated total RNA (1 μg) was reverse transcribed using an antisense primer (5’-AAC GGC TGC TGC CAG CCA AAT AGC ATC CAA-3’) complementary to exon 6 of the *TMEM38B* transcript (NM_018112.2) and murine leukemia virus (MuLV) reverse transcriptase (Life Technologies). Subsequent PCR used the same antisense primer and a sense primer corresponding to exon 2 sequence (5’-GGA GCA GCT GCA TTG GCA TGG AAG AAT CCT-3’). Reaction products were electrophoresed on 1.5% agarose gels and visualized with ethidium bromide. Alternatively, spliced transcripts were purified by extraction of individual gel bands using the QIAquick gel extraction kit (Qiagen), followed by cloning into plasmids (TOPO TA cloning kit, Life Technologies) and sequencing using vector-specific primers. To quantitate splice products, RT-PCR reactions were repeated in the presence of 2.5 μCi of 111TBq/mmol [α-^32^P] dCTP for 20 or 25 cycles. PCR products were electrophoresed on 6% TBE gels and individual bands were quantitated by densitometry of autoradiographs.

For *XBP1* splicing, 1 μg of DNase-treated total RNA was reverse-transcribed using the SuperScript One-Step RT-PCR System (Life Technologies). The resulting cDNA was used as a template for PCR amplification across the region of the *XBP1* cDNA containing the intronic target of IRE1α ribonuclease activity using 15 μM sense (5’-TCA GCT TTT ACG AGA GAA AAC TCA TGG CCT-3’) and antisense primers (5’-AGA ACA TGA CTG GGT CCA AGT TGT CCA GAA-3’). Following a 30 min incubation at 50°C, reactions were cycled 30 times at 94°C, 60°C and 72°C for 30 sec at each temperature. Reaction products were electrophoresed on 6% TBE gels (Life Technologies) and visualized by ethidium bromide staining.

### Western blot analysis

Fibroblast cultures were lysed in RIPA buffer (150 mM NaCl, 1% NP-40, 0.5% Na-deoxycholate, 0.1% SDS, 50 mM Tris, pH 7.4) supplemented with protease inhibitor cocktail (Sigma-Aldrich). Protein concentration was determined using the BCA Protein Assay Kit (Thermo Scientific). Samples (20 μg protein) were subjected to SDS-PAGE on 4–15% acrylamide gels (BioRad) under denaturing conditions and transferred to nitrocellulose membranes. The membranes were blocked overnight in 5% non-fat milk in TBST. After washing in TBST, membranes were incubated overnight at 4°C in TBST containing 2.5% non-fat milk and primary antibody (diluted 1:500–1000). After washing in 1X TBST, membranes were incubated with the corresponding IRDye infrared secondary antibody (diluted 1:10,000) (LI-COR Biosciences). Proteins were visualized using an Odyssey Infrared Imaging System (LI-COR Biosciences) and quantitated using the Odyssey 3.0.30 software with normalization to actin levels. The primary antibodies used include rabbit anti-TMEM38B (Thermo Fisher; PA5-20859), mouse anti-TMEM38A (Abnova; H00079041-B01P), rabbit anti-IP3R1 (Novus Biologicals; NB120-5908), rabbit anti-SERCA2 (Novus Biologicals; NBP1-59203), rabbit anti-GRP78 (Abcam; ab32618), mouse anti-FKBP65 (Abnova; H00060681-M13), rabbit anti-cyclophilin B/PPIB (Abcam; ab16045), rabbit anti-PLOD1 (Abgent; AP12656c), rabbit anti-PLOD2 (Proteintech; 21214-1-AP), rabbit anti-ATF4 (Proteintech; 10835-1-AP), mouse anti-ATF6 (Active Motif; 40962), and mouse anti-PDI (Novus Biologicals; H00005034-B01P). Rabbit anti-α1(I) C-telopeptide (LF68) was a generous gift from Larry Fisher, NIDCR, NIH. Rabbit (Santa Cruz Biotechnology; sc-1616-R) and mouse (Sigma-Aldrich; A5441) anti-ACTIN antibodies were used as loading controls. Western analyses were performed in duplicate on independent cultures to ensure reproducibility.

### [Ca^2+^]_i_ measurements

For determination of [Ca^2+^] flux into the cytoplasm, normal control and proband fibroblasts and osteoblasts were cultured in 96-well plates for 3 days prior to loading with 5 μM Fura-2AM (Life Technologies) in HBSS for 30 min at 37°C in 5% CO_2_. The Ca^2+^ transients were recorded as the 340/380 nm ratio (R) of the resulting 510-nm emissions using a Mithras LB 940 plate reader (Berthold Technologies), as previously described [61]. Intracellular [Ca^2+^] was calculated using the equation [Ca^2+^]_i_ = K_d_ (R-R_min_)/(R_max_-R)(F^380^_max_/F^380^_min_), where R_min_ is the ratio at zero [Ca^2+^], R_max_ is the ratio when Fura-2 is completely saturated with Ca^2+^, F^380^_min_ is the fluorescence at 380 nm for zero [Ca^2+^], and F^380^_max_ is the fluorescence at saturating [Ca^2+^] and K_d_ = 224 nM [62]. To stimulate Ca^2+^ release, 200 μM of ATP or 2 μg/ml ionomycin was injected into cultures using the Mithras 940. For inhibition experiments, cells were incubated for 30 min before analysis with either 100 μM 2-APB to block IP_3_R, or 100 nM thapsigargin for SERCA ER Ca^2+^ pump inactivation.

Endoplasmic reticulum luminal free [Ca^2+^] was quantified using D1ER, a cameleon class calcium FRET sensor containing YFP and CFP, as previously described [25,26]. Normal and proband fibroblasts (5 x 10^5^ cells) were transfected with 2 μg D1ER or with 1 μg of pmaxGFP plasmid by electroporation using a 4D-Nucleofector system (Lonza), seeded into covered chamber slides and cultured at 37°C in 5% CO_2_ for 48 hr. Following a 30 min incubation in Ca^2+^-free HBSS, cells were treated with 1 mM ATP to evoke transient depletion of ER calcium. Cells were then treated with 5 μM ionomycin (Sigma) and 3 mM EGTA (Sigma) to obtain a minimum ER calcium signal reference value. Images were obtained on a Zeiss 510 laser scanning microscope using a 20x 0.8 NA objective. D1ER was excited at 405 nm. CFP emission was collected at 420–480 nm and the FRET signal was collected at 505–550 nm. Ratios were calculated in ImageJ (v1.50i, NIH, USA) by first subtracting the background using the Rolling Ball plugin (50 pixel radius, paraboloid) and filtering using a Gaussian blur (2 pixel radius) for each image and then dividing the FRET image by the CFP image. The ratio was then normalized to the average ratio obtained 6 frames before ATP application (R_0_). R_min_ was determined 1 min after ionomycin application from the new stable baseline using a 6-frame average. Calcium signal changes between baseline (before ATP treatment) and depletion (after ionomycin treatment) were evaluated using ΔR = R_min_/R_0_−1.

### Biochemical analysis of type I collagen

Steady-state collagen analysis was performed as previously described [63]. Normal control and proband fibroblasts or osteoblasts were grown to confluence in 6-well culture dishes. Cells were incubated with 437.5 μCi/ml of 3.96 TBq/mmol L-[2,3,4,5-^3^H] proline for 18 h prior to collection. Procollagens were precipitated overnight at 4°C with 176 mg/ml ammonium sulfate, pelleted by centrifugation at 37,000 x g, and digested with pepsin (50 μg/ml) for 4 h. Collagen alpha chains were separated on 6% SDS-urea-polyacrylamide gels and visualized by autoradiography.

For analysis of collagen modification, confluent cell cultures were stimulated for collagen synthesis in DMEM containing 0.1% FBS and 100 μg/ml ascorbate for three days, with daily collection. Collected medium was buffered with 100 mM Tris-HCl, pH 7.4, and protease inhibitors were added to the following final concentrations: 25 mM EDTA, 0.02% NaN_3_, 1 mM phenylmethylsulfonylfluoride, 5 mM benzamidine, and 10 mM N-ethylmaleimide. Procollagens were then precipitated with 176 mg/ml ammonium sulfate overnight at 4°C and pelleted by centrifugation at 16,000 x g for 2 h. Procollagens were resuspended in 0.5 M acetic acid and digested with 0.1 mg/ml pepsin at 4°C overnight. Selective salt precipitation of type I collagen with 0.9 M NaCl in 0.5 M acetic acid was performed twice, followed by dialysis against 5mM acetic acid overnight and lyophilization for further analyses.

Differential Scanning Calorimetry (DSC) scans were performed as previously described [64]. Thermograms were recorded in 0.2 M sodium phosphate, 0.5 M glycerol, pH 7.4, from 10 to 50°C at 0.125°C/min heating rates in a Nano III DSC instrument (Calorimetry Sciences Corporation).

Amino acid analysis to quantify total 4-hydroxyproline, proline, 5-hydroxylysine and lysine content of type I collagen was performed by high-pressure liquid chromatography (AIBiotech). For determination of modification of specific residues pro-alpha chains of type I procollagen were resolved by SDS-PAGE, followed by in-gel trypsin digestion for analysis of targeted peptides. Electrospray mass spectrometry was performed using an LTQ XL ion-trap mass spectrometer equipped with in-line liquid chromatography (Thermo Scientific) using a C4 5 μm capillary column (300 μm × 150 mm; Higgins Analytical RS-15M3-W045). The LC mobile phase consisted of buffer A (0.1% formic acid in MilliQ water) and buffer B (0.1% formic acid in 3:1 acetonitrile:n-propanol v/v). The LC sample stream was introduced into the mass spectrometer by electrospray ionization (ESI) with a spray voltage of 4 kV. Proteome Discoverer software (Thermo Scientific) was used for peptide identification using the NCBI protein database.

### Procollagen chain association

Procollagen chain incorporation was followed as previously described [65]. Procollagen alpha chains were labeled with a pulse of 140 μCi/ml of 3.96 TBq/mmol L-[2,3,4,5-^3^H] proline for 80 min, then chased with DMEM containing 10% serum, 50 μg/ml ascorbic acid and 10 mM proline for the time intervals specified. Samples were ethanol precipitated, separated on 5% SDS-Urea-polyacrylamide gels and visualized by autoradiography.

### Collagen synthesis and secretion kinetics

For pulse-chase assays, performed in triplicate, normal control and proband fibroblasts or osteoblasts were grown to confluence in 6-well culture dishes [66]. For each cell line, two wells were used for cell counts. Cells were labeled for 4 h with 1.75 μCi [^14^C]-proline and then chased with fresh medium containing 2 mM cold proline. Medium and cell layer procollagens were harvested at the indicated times, digested with pepsin and precipitated with 2M NaCl. Samples were loaded for equivalent cell number on 3–8% Tris-acetate gels. Collagen alpha chains were quantitated by densitometry and expressed as the percent secreted at each time point, as determined by (media)/(cell + media) x 100.

Alternatively, cells were grown to confluence in 75 cm^2^ culture flasks and incubated in phenol red-free DMEM (Life Technologies) containing 0.1% FBS and 100 μg/ml ascorbic to generate conditioned media. After 24 h, media was collected, supplemented with protease inhibitor cocktail (Sigma-Aldrich), and concentrated using Amicon Ultra-4 centrifugal filter devices (Millipore). Total collagen derived from each flask was quantitated using the Sircol Soluble Collagen Assay (Biocolor Ltd) according to the manufacturer’s instructions. Immediately following media collection, cultures were trypsinized and collected for determination of cell count from each flask. Data is expressed as amount of collagen secreted per cell per hour and were generated from 3 fibroblast and osteoblast cultures per proband, 3 replicates of the normal osteoblast control cultures, and 6 normal control fibroblast cultures.

### Procollagen pericellular processing

Processing of procollagens secreted by fibroblasts was examined by labeling confluent cells with 260 μCi/ml of 3.96 TBq/mmol L-[2,3,4,5-^3^H] proline for 24 h and then chased with DMEM containing 2 mM non-radioactive proline and 10% fetal bovine serum. Media from independent wells were harvested at 24-h intervals over a 5-day period as previously described, precipitated with ammonium sulfate, electrophoresed on 6% polyacrylamide-urea-SDS gels, and visualized by autoradiography [67].

### Collagen matrix deposition

Normal control and proband primary fibroblasts were grown to confluence and stimulated every other day for 14 days with fresh DMEM containing 10% FBS and 100 μg/ml ascorbic acid, as described [68], and then labeled for 24 h with 437.5 μCi/ml L-[2,3,4,5-^3^H] proline. Matrix collagens were sequentially extracted at 4°C, with neutral salt for newly incorporated collagen, acetic acid for collagens with acid-labile cross-links, and pepsin digestion for collagens with mature cross-links [69]. All fractions were electrophoresed on 6% polyacrylamide-urea-SDS gels. Samples were loaded for equivalent densitometry signal, and the total signal for each fraction calculated by adjusting the signal by the total volume of that fraction.

### Statistical analysis

Data were analyzed using an unpaired Student *t*-test with two-tailed analysis and considered statistically significant at p < 0.05.

## Supporting Information

S1 Fig *TMEM38B* Bedouin founder mutation results in minimal in-frame transcripts due to alternative splicing. **(A)** Characterization of alternative splicing in P1 fibroblasts, including 4 out-of-frame and 1 in-frame transcript. Arrows represent positions of primers used for RT-PCR amplification. (*) represents positions of premature termination codons in alternative transcripts. **(B)** Quantitation of splice forms from Proband 1 fibroblast RNA. Densitometric analysis of RT-PCR products, amplified for 20 or 25 cycles, demonstrates the in-frame variant (*, 368 bp) is 8% of the signal of the expected 639 bp product from normal control fibroblast mRNA.
(TIF)

S2 Fig Relative expression of TRIC isoforms and ER calcium release channels. **(A)** Relative mRNA transcript levels of *TMEM38A* and *TMEM38B* in normal and proband fibroblasts and osteoblasts. Transcript levels were normalized to three reference genes (*ACTB*, *B2M* and *GAPDH*) and are expressed relative to normal control fibroblast *TMEM38B* transcripts. **(B)** Levels of *RYR1* and *ITPR1* transcripts in normal and proband fibroblasts and osteoblasts, versus normal fibroblast control *ITPR1* expression. Relative expression was determined using the comparative CT method (ΔΔCt, http://www3.appliedbiosystems.com/cms/groups/mcb_support/documents/generaldocuments/cms_042380.pdf). **, p < 0.01.
(TIF)

S3 Fig Decreased Ca^2+^ mobilization in TRIC-B deficient cells. **(A)** Ionomycin-stimulated Ca^2+^ release to deplete all intracellular stores, followed by ATP-stimulated IP_3_R-mediated Ca^2+^ release, demonstrates absence of replenishment of ER Ca^2+^ stores in P1 fibroblasts. **(B)** Decreased Ca^2+^ release and delayed return to baseline following ionomycin-stimulated Ca^2+^ release suggest global dysregulation of intracellular Ca^2+^ homeostasis. Ca^2+^ efflux was completely blocked in the presence of the IP_3_R inhibitor 2-APB (dashed lines).
(TIF)

S4 Fig Altered ER [Ca^2+^] does not delay cyclophilin B-mediated collagen folding. Assay for intracellular folding of type I collagen in Proband 1 (P1) fibroblast cultures versus normal control (C) fibroblasts. The equivalent rate of trypsin-resistant collagen alpha chains suggests normal functioning of cyclophilin B-mediated peptidyl-prolyl *cis-trans* isomerization, the rate-limiting step in collagen folding. Data represents the average from three experiments.
(TIF)

S5 Fig Pulse-chase analysis of pericellular procollagen processing. Procollagens secreted by Proband 1 (OI) and normal control (C) fibroblasts were collected at 1-day intervals and separated by SDS-Urea PAGE to follow their processing by pericellular propeptidases. There is an increase in the fraction of type I procollagen molecules that are resistant to amino propeptide removal (pN-α1(I) and pN-α2(I), denoted by *) in proband cell cultures at all timepoints.
(TIF)

S6 Fig Pulse-chase analysis of collagen secretion. Type I collagen secretion kinetics are equivalent in Proband 1 (P1) and normal control (C) fibroblast cultures. However, comparison of the quantity of collagen in cell and media fractions at each timepoint demonstrate a nearly 40% decrease in the amount of collagen in proband samples.
(TIF)

S7 Fig Amount of type I collagen deposited into extracellular matrix in culture is decreased in the absence of TRIC-B. Normal control (C) and Proband 1 (P1) fibroblasts were grown to confluence and stimulated for collagen synthesis for 2 weeks with ascorbate. Following a 24-hr pulse of labeled collagen, secreted collagens were collected and extracellular matrix was serially extracted with neutral salt (NS), acetic acid (AA) for immaturely crosslinked and pepsin (P) for maturely crosslinked collagen isolation. Samples were loaded for type I collagen balance. Note the difference in the pepsin-soluble fraction of P1 matrix versus normal control matrix; the relative increase in P1 type III collagen (α1(III)_3_) suggests a decrease in the amount of type I collagen capable of matrix incorporation.
(TIF)

S8 Fig Validation of normal primary fibroblast control cell lines. The relative expression of genes of interest in three independent normal control fibroblast cell lines (NL C1, C2 and C3) was evaluated by real-time RT-PCR. Expression levels were compared to values obtained for pooled cDNA (NL pool) derived from each control cell line. Only *COL1A1* expression varied significantly among the 3 normal control cell lines. *, p < 0.05.
(TIF)
