# Supplementary figures and images for: Absence of the ER Cation Channel TMEM38B/TRIC-B Disrupts Intracellular Calcium Homeostasis and Dysregulates Collagen Synthesis in Recessive Osteogenesis Imperfecta
Source: PLoS Genet. 2016 Jul 21;12(7):e1006156. doi: 10.1371/journal.pgen.1006156 (PMC4956114; doi:10.1371/journal.pgen.1006156)

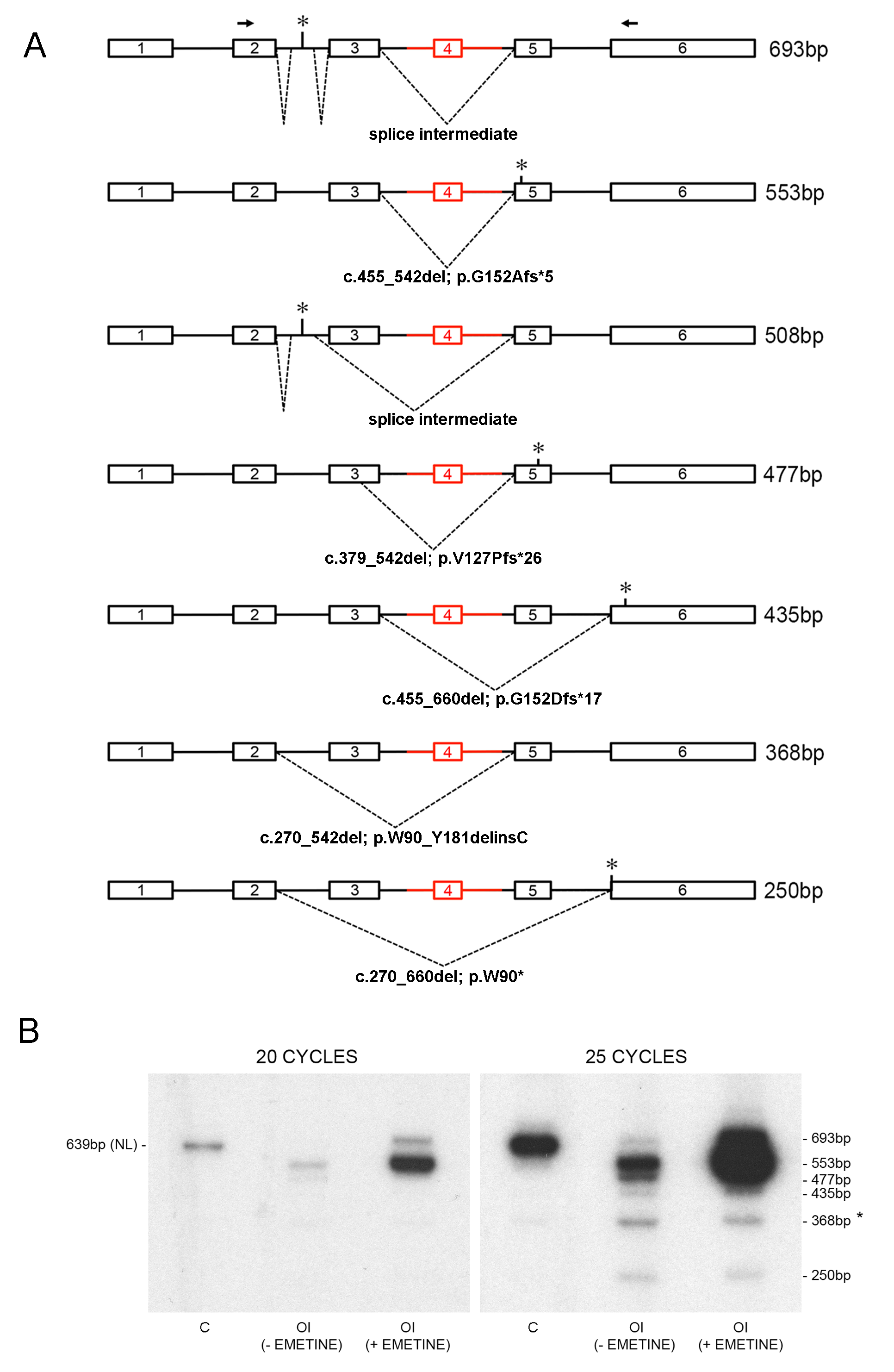

Supplement: S1 Fig — (A) Characterization of alternative splicing in P1 fibroblasts, including 4 out-of-frame and 1 in-frame transcript. Arrows represent positions of primers used for RT-PCR amplification. (*) represents positions of premature termination codons in alternative transcripts. (B) Quantitation of splice forms from Proband 1 fibroblast RNA. Densitometric analysis of RT-PCR products, amplified for 20 or 25 cycles, demonstrates the in-frame variant (*, 368 bp) is 8% of the signal of the expected 639 bp product from normal control fibroblast mRNA. (TIF) [file pgen.1006156.s001.tif]

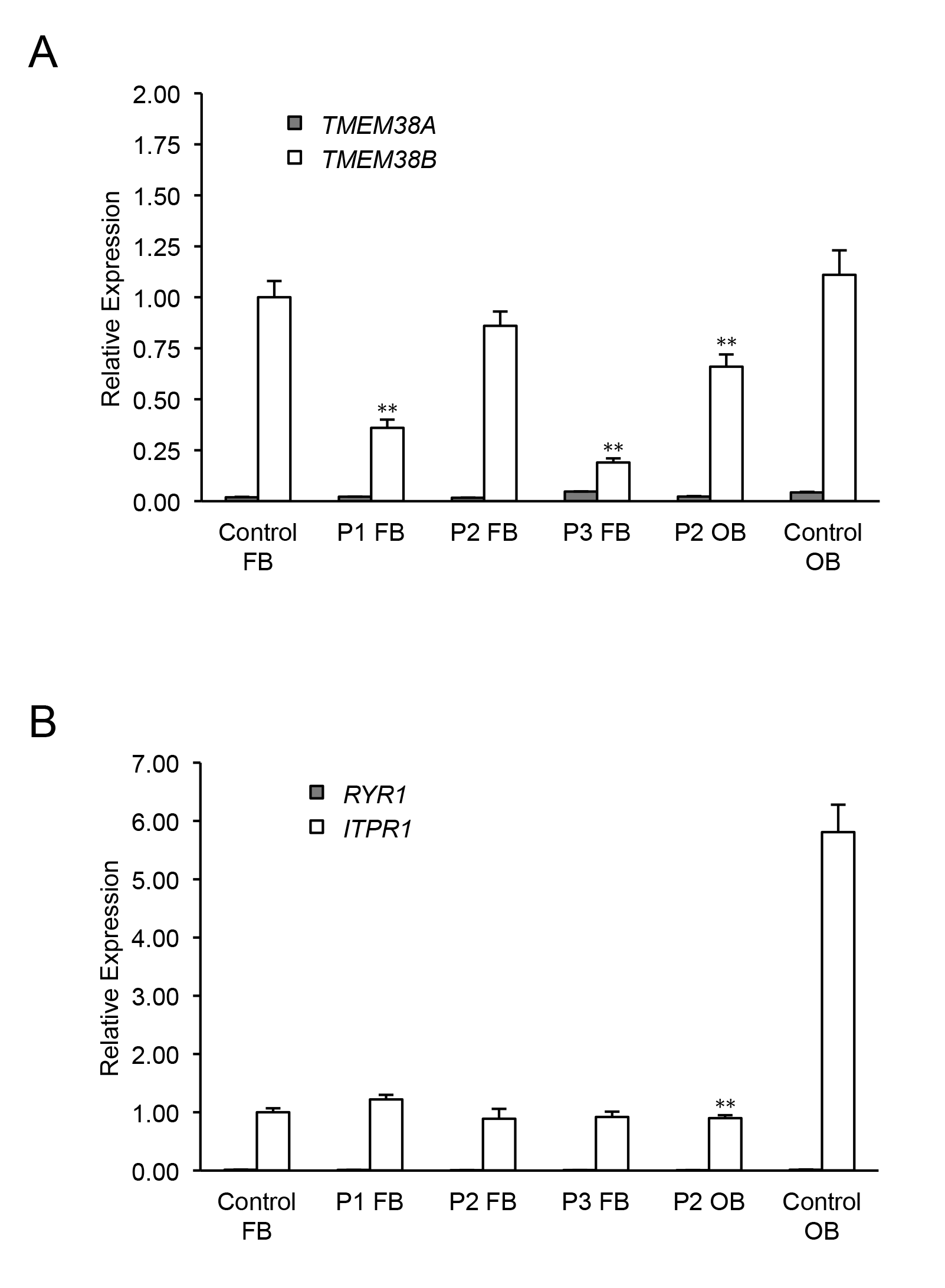

Supplement: S2 Fig — (A) Relative mRNA transcript levels of TMEM38A and TMEM38B in normal and proband fibroblasts and osteoblasts. Transcript levels were normalized to three reference genes (ACTB, B2M and GAPDH) and are expressed relative to normal control fibroblast TMEM38B transcripts. (B) Levels of RYR1 and ITPR1 transcripts in normal and proband fibroblasts and osteoblasts, versus normal fibroblast control ITPR1 expression. Relative expression was determined using the comparative CT method (ΔΔCt, http://www3.appliedbiosystems.com/cms/groups/mcb_support/documents/generaldocuments/cms_042380.pdf). **, p < 0.01. (TIF) [file pgen.1006156.s002.tif]

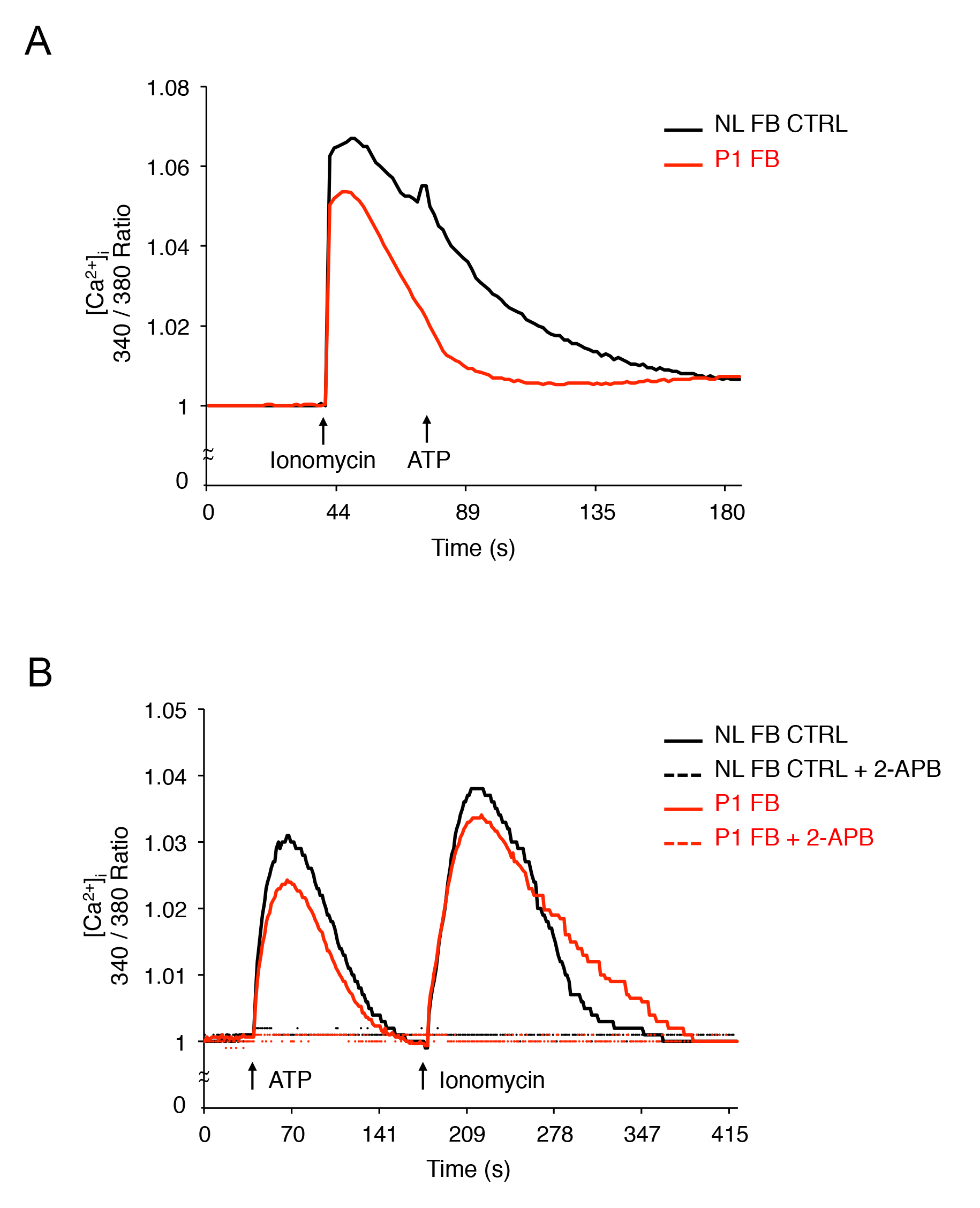

Supplement: S3 Fig — (A) Ionomycin-stimulated Ca2+ release to deplete all intracellular stores, followed by ATP-stimulated IP3R-mediated Ca2+ release, demonstrates absence of replenishment of ER Ca2+ stores in P1 fibroblasts. (B) Decreased Ca2+ release and delayed return to baseline following ionomycin-stimulated Ca2+ release suggest global dysregulation of intracellular Ca2+ homeostasis. Ca2+ efflux was completely blocked in the presence of the IP3R inhibitor 2-APB (dashed lines). (TIF) [file pgen.1006156.s003.tif]

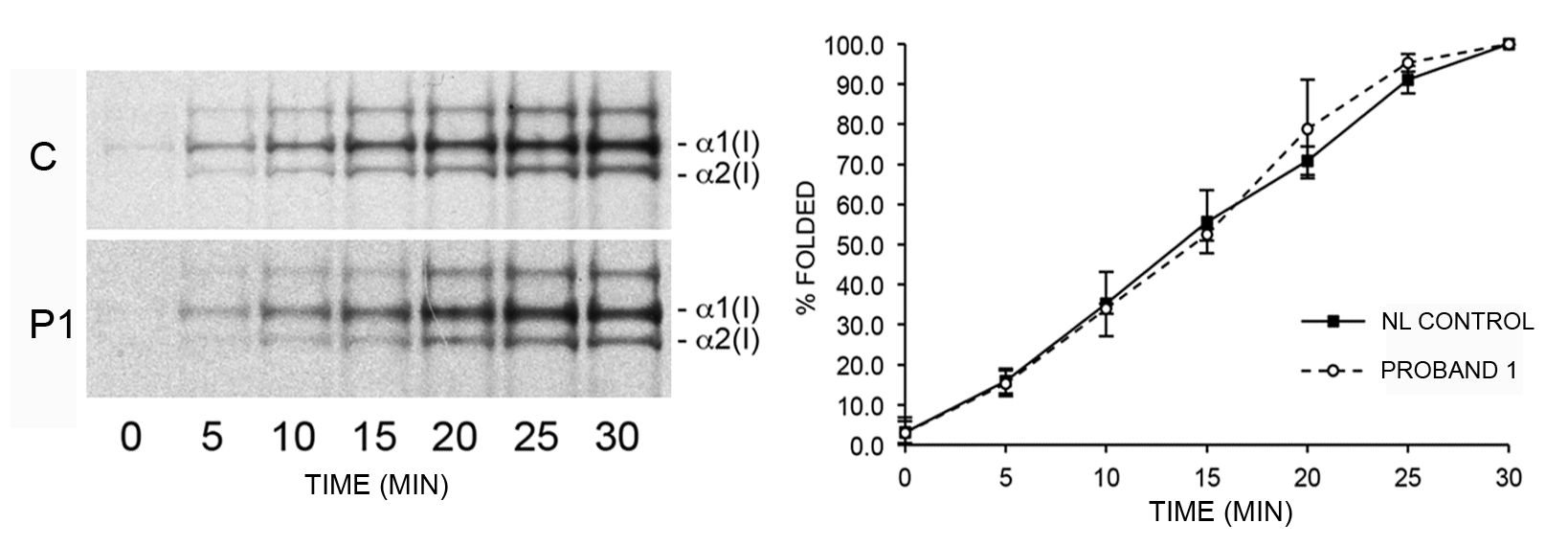

Supplement: S4 Fig — Assay for intracellular folding of type I collagen in Proband 1 (P1) fibroblast cultures versus normal control (C) fibroblasts. The equivalent rate of trypsin-resistant collagen alpha chains suggests normal functioning of cyclophilin B-mediated peptidyl-prolyl cis-trans isomerization, the rate-limiting step in collagen folding. Data represents the average from three experiments. (TIF) [file pgen.1006156.s004.tif]

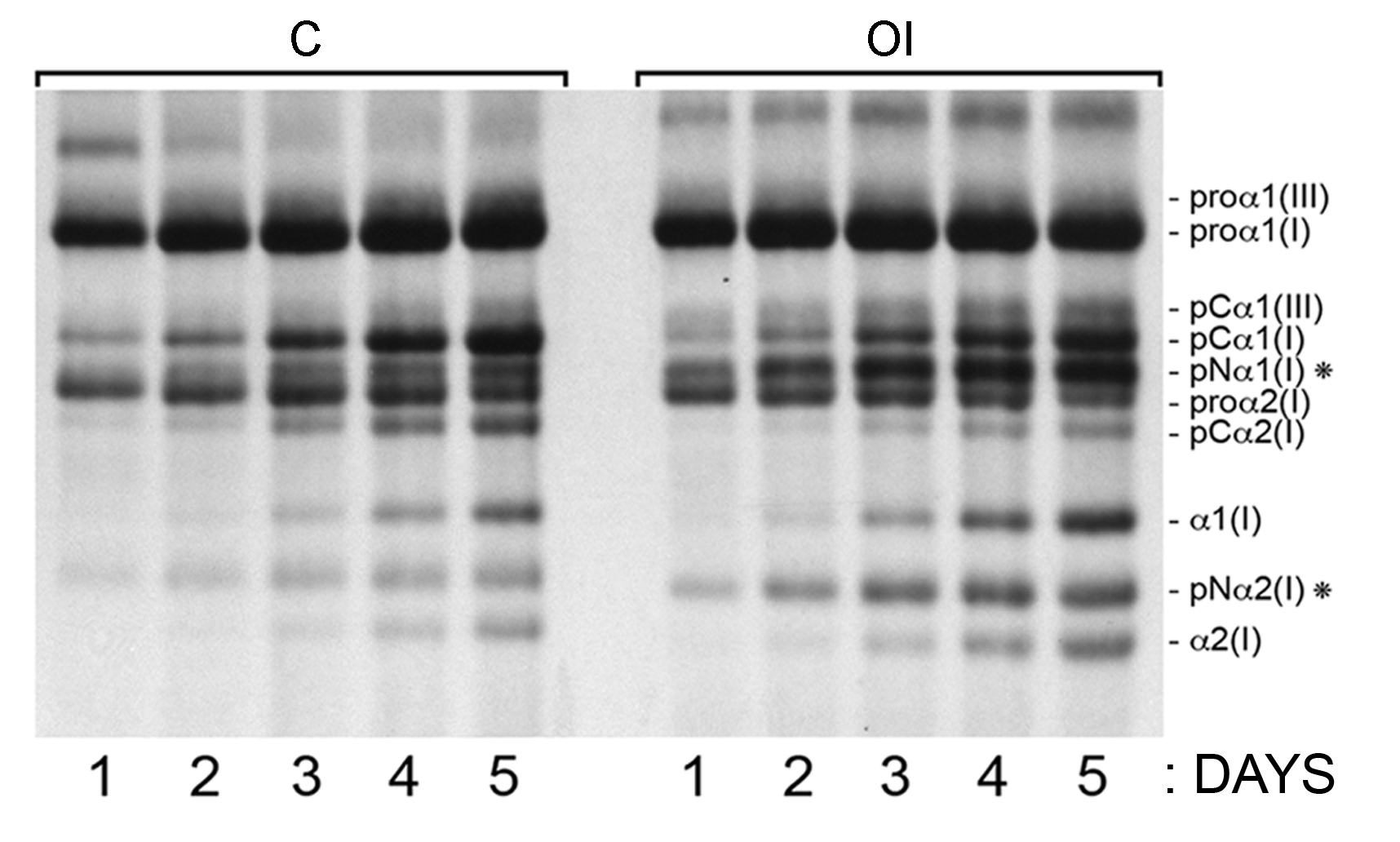

Supplement: S5 Fig — Procollagens secreted by Proband 1 (OI) and normal control (C) fibroblasts were collected at 1-day intervals and separated by SDS-Urea PAGE to follow their processing by pericellular propeptidases. There is an increase in the fraction of type I procollagen molecules that are resistant to amino propeptide removal (pN-α1(I) and pN-α2(I), denoted by *) in proband cell cultures at all timepoints. (TIF) [file pgen.1006156.s005.tif]

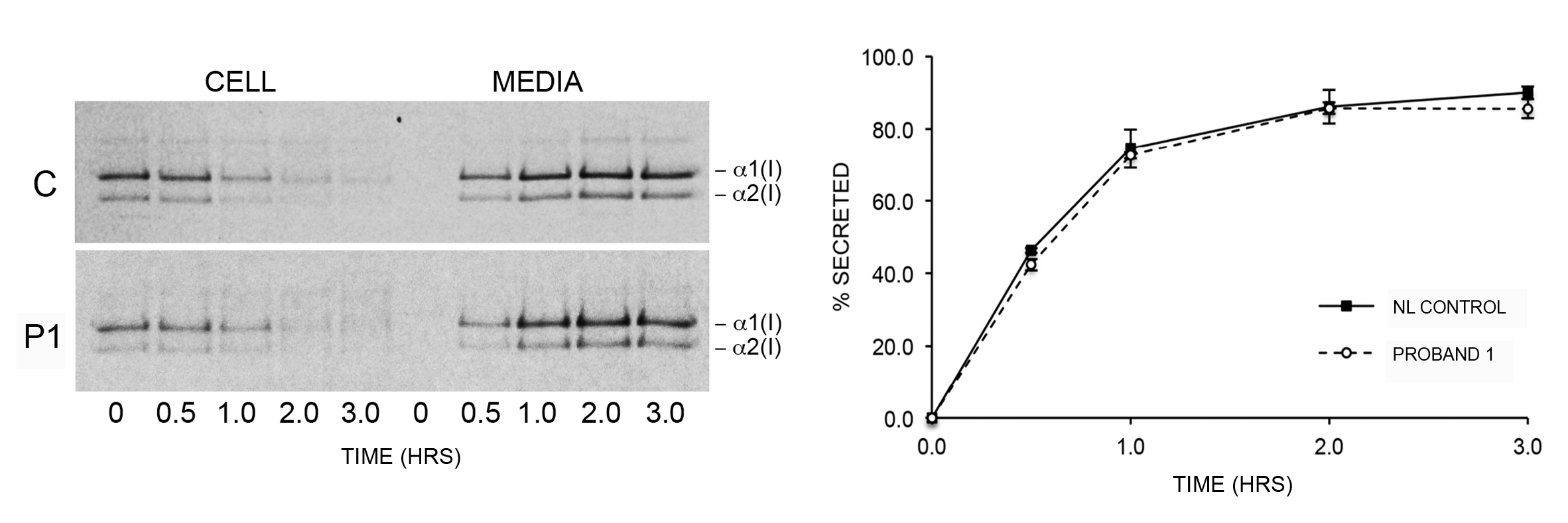

Supplement: S6 Fig — Type I collagen secretion kinetics are equivalent in Proband 1 (P1) and normal control (C) fibroblast cultures. However, comparison of the quantity of collagen in cell and media fractions at each timepoint demonstrate a nearly 40% decrease in the amount of collagen in proband samples. (TIF) [file pgen.1006156.s006.tif]

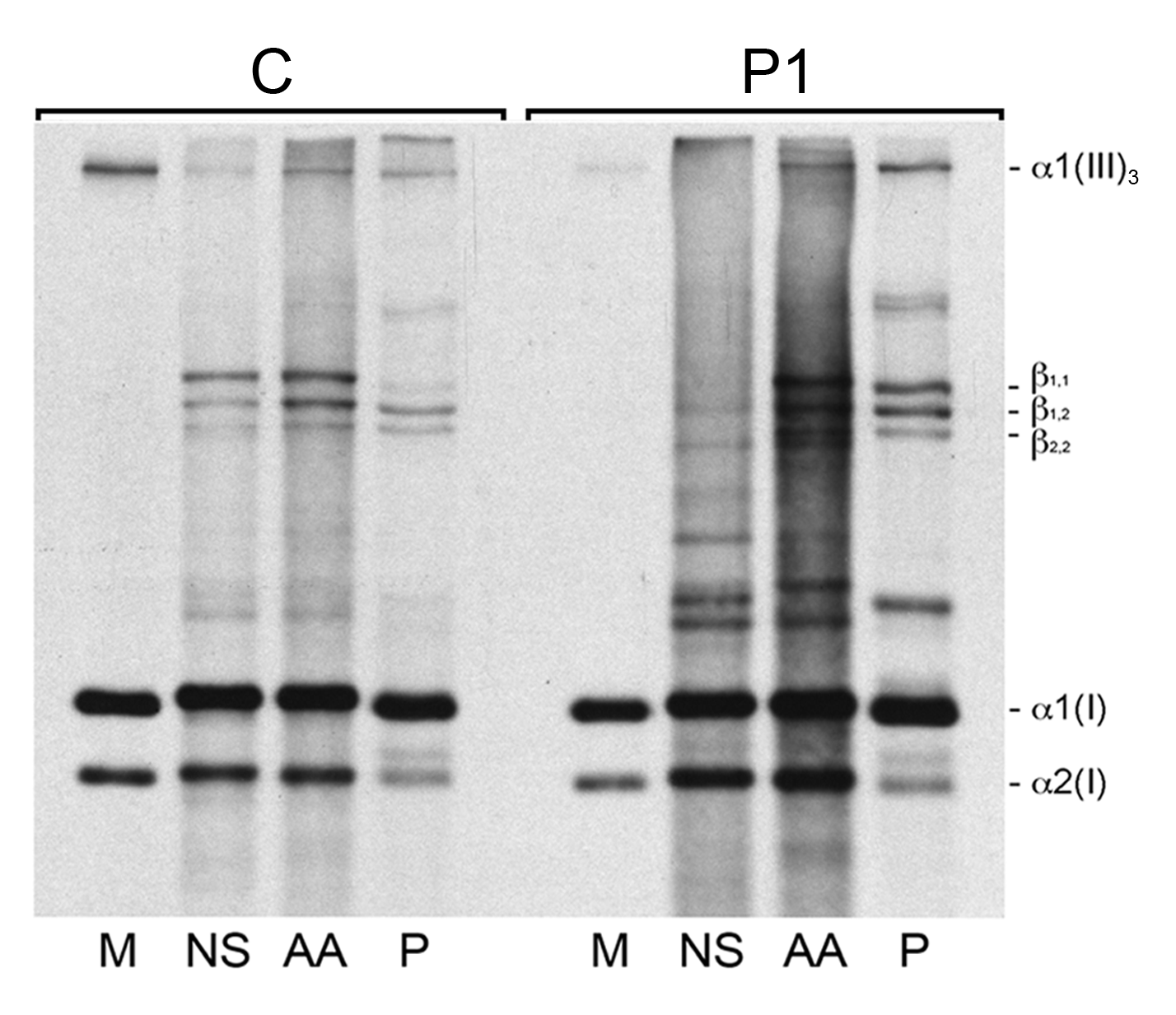

Supplement: S7 Fig — Normal control (C) and Proband 1 (P1) fibroblasts were grown to confluence and stimulated for collagen synthesis for 2 weeks with ascorbate. Following a 24-hr pulse of labeled collagen, secreted collagens were collected and extracellular matrix was serially extracted with neutral salt (NS), acetic acid (AA) for immaturely crosslinked and pepsin (P) for maturely crosslinked collagen isolation. Samples were loaded for type I collagen balance. Note the difference in the pepsin-soluble fraction of P1 matrix versus normal control matrix; the relative increase in P1 type III collagen (α1(III)3) suggests a decrease in the amount of type I collagen capable of matrix incorporation. (TIF) [file pgen.1006156.s007.tif]

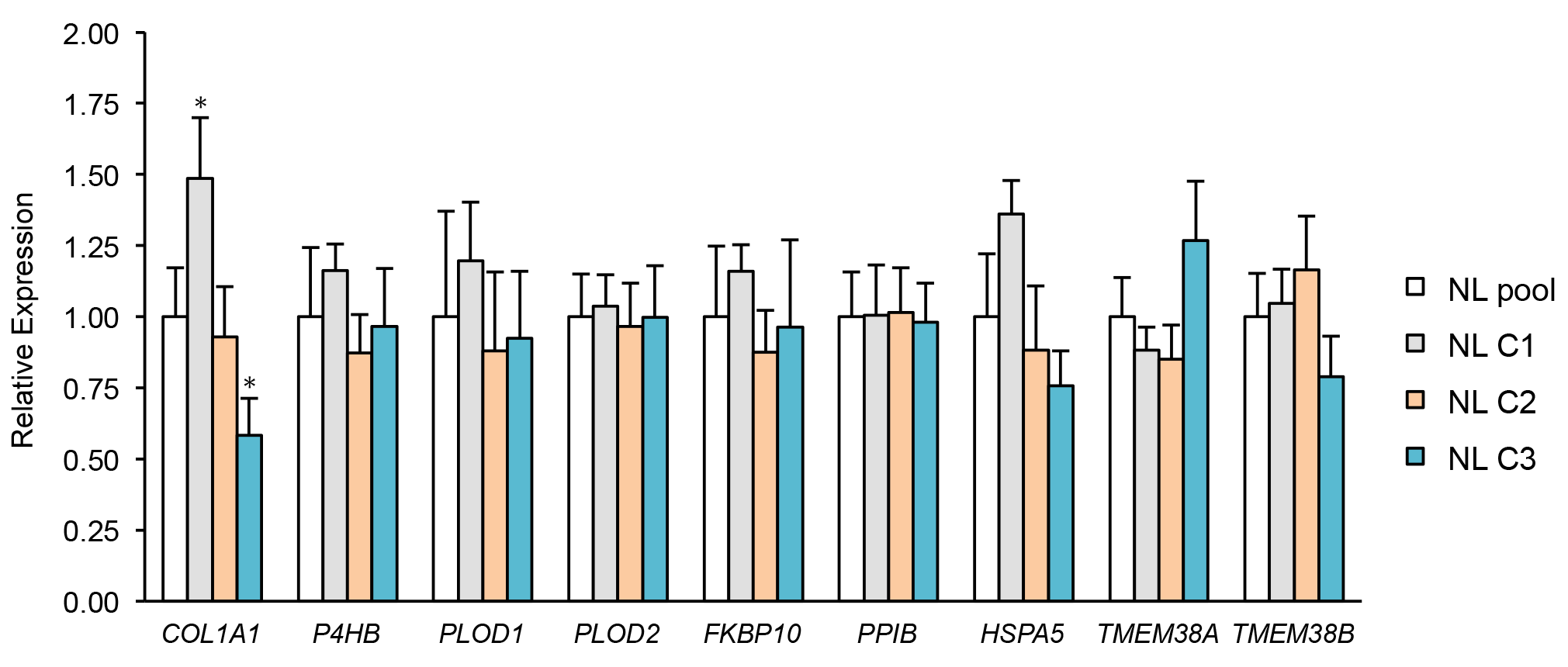

Supplement: S8 Fig — The relative expression of genes of interest in three independent normal control fibroblast cell lines (NL C1, C2 and C3) was evaluated by real-time RT-PCR. Expression levels were compared to values obtained for pooled cDNA (NL pool) derived from each control cell line. Only COL1A1 expression varied significantly among the 3 normal control cell lines. *, p < 0.05. (TIF) [file pgen.1006156.s008.tif]
